# Breaking the barrier: from biosynthetic inhibition to multidimensional modulation of the mycobacterial cell wall in tuberculosis therapy

**DOI:** 10.3389/fphar.2026.1863735

**Published:** 2026-06-15

**Authors:** Pan Li, Yanmei Liao, Lingli Zhu, Min Zhang, Yuhan Wang, Li Liao, Tao Wu, Xueliang Chen, Yingyong Xia, Xue Han

**Affiliations:** 1 Department of Pharmacy, Chengdu Qingbaijiang District People’s Hospital, Chengdu, Sichuan, China; 2 Department of Pharmacy, Public Health Clinical Center of Chengdu, Chengdu, Sichuan, China; 3 School of Pharmacy, Chengdu Medical College, Chengdu, Sichuan, China

**Keywords:** anti-tuberculosis drug discovery, cell wall biosynthesis, DprE11, drug resistance, *Mycobacterium tuberculosis*

## Abstract

Tuberculosis remains a leading cause of global mortality, with the rise of multidrug resistant and extensively drug-resistant *Mycobacterium tuberculosis* strains highlighting an urgent need for novel therapeutics. The mycobacterial cell wall has long served as a validated drug target, owing to its unique architecture comprising arabinogalactan, peptidoglycan, and mycolic acids. This review provides a comprehensive overview of recent advances in cell wall targeting anti tuberculosis drug discovery, encompassing both established and emerging targets within the major biosynthetic pathways. Beyond conventional enzymatic inhibition, a multidimensional framework is explored that includes disrupting cell wall integrity through alternative mechanisms, interfering with energy metabolism and regulatory networks, blocking the secretion and transport of cell wall components, harnessing host-directed therapies and antimicrobial peptides, and leveraging nanotechnology-based delivery systems. By integrating discoveries across these diverse fronts, this review highlights evolving strategies and offers a forward looking perspective on the development of ultra short, highly effective, and resistance proof tuberculosis regimens.

## 1 Introduction

Tuberculosis (TB), caused by *Mycobacterium tuberculosis* (Mtb), remains one of the most devastating infectious diseases worldwide. According to the World Health Organization, approximately 10.7 million new cases and 1.23 million deaths were reported in 2024, with TB reemerging as the leading cause of death from a single infectious agent after the COVID-19 pandemic (Zhang Y. et al., 2025; World Health Organization, 2025). Standard first-line regimens comprising rifampicin, isoniazid, pyrazinamide, and ethambutol are effective against drug-susceptible TB. However, their prolonged treatment duration of six to 9 months, coupled with poor patient adherence, has contributed to the alarming rise of multidrug-resistant (MDR) and extensively drug-resistant (XDR) strains. Moreover, only a limited number of novel anti TB drugs, including bedaquiline, delamanid, and pretomanid, have been approved in recent decades, highlighting the urgent need for new therapeutic strategies with distinct mechanisms of action to overcome resistance and shorten treatment duration (Zhang Y. et al., 2025; Diab et al., 2025).

The mycobacterial cell wall, characterized by its unique and complex architecture, has long been a validated target for anti TB drug discovery. Its three core structural layers, namely, an outer mycolic acid layer, an intermediate arabinogalactan polysaccharide, and an inner peptidoglycan layer, provide not only structural integrity and protection against environmental stress but also contribute critically to Mtb pathogenicity and intrinsic drug resistance (Abrahams and Besra, 2016). Historically, targeting cell wall biosynthesis has proven highly successful, as exemplified by isoniazid, which inhibits InhA in mycolic acid synthesis, and ethambutol, which targets arabinosyltransferases in arabinogalactan assembly. However, the increasing prevalence of drug resistance, driven largely by mutations in target encoding genes such as inhA, katG, and rpoB, has exposed the limitations of strategies focused solely on inhibiting biosynthetic enzymes (Pflégr et al., 2021).

Whether mere inhibition of cell wall synthesis is sufficient to achieve rapid and durable bacterial eradication in the era of escalating drug resistance remains an open question. Thus, a multidimensional approach is necessary. Beyond the conventional paradigm of blocking construction, recent advances have unveiled a spectrum of innovative strategies that may be conceptualized as subverting the guardians. These approaches target not only the biosynthetic machinery but also the regulatory networks governing cell wall homeostasis, the transport systems responsible for delivering essential building blocks, the energy metabolism that powers these processes, and the host immune response to enhance bacterial clearance (Kotliarova et al., 2024). In parallel, novel drug delivery systems such as nanoparticles and niosomes have been developed to overcome the formidable permeability barrier of the mycobacterial cell wall, enabling more effective delivery of both existing and new therapeutic agents (El-Ridy et al., 2015; Khatak et al., 2020).

**FIGURE 1:**
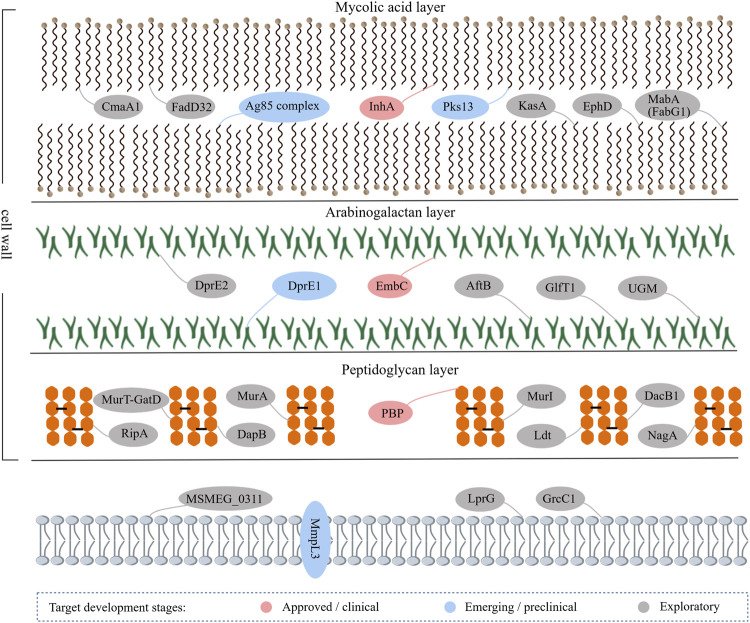
Mycobacterial cell wall architecture and key drug targets. The cell wall comprises three core layers: outer mycolic acid layer (adapted from (Dulberger et al., 2019; Daffé and Marrakchi, 2019)), intermediate arabinogalactan layer, and inner peptidoglycan layer, overlying the plasma membrane. Key enzymes and transporters are shown at their approximate sites of action. The corresponding gene names are: inhA (InhA), embC (EmbC), dprE1 (DprE1), mmpL3 (MmpL3), pks13 (Pks13), kasA (KasA), mabA (MabA), glfT1 (GlfT1), ugm (UGM), aftB (AftB), murA (MurA), murT/gatD (MurT-GatD), dapB (DapB), murI (MurI), ponA1 (PBP), ldtMt1-3 (Ldt), ripA (RipA), nagA (NagA), lprG (LprG), grcC1 (GrcC1). Color code for target development stages: red, approved/clinical; blue, emerging/preclinical; gray, exploratory.

The architecture of the Mtb cell wall and the key targets involved in its biosynthesis and transport are shown in Figure 1 (Jiang et al., 2025). This review provides a comprehensive overview of cell wall targeting strategies for tuberculosis treatment. It summarizes the key biosynthetic pathways of arabinogalactan, peptidoglycan, and mycolic acids, along with associated inhibitors, including both established drugs and clinical candidates. Emerging concepts beyond direct inhibition are explored, including disruption of cell wall integrity, interference with energy metabolism and regulatory circuits, blockade of secretion and transport systems, and application of host directed therapies and antimicrobial peptides (AMPs). The potential of nanotechnology based delivery systems to enhance drug penetration and efficacy is also discussed. This review offers a forward looking perspective on exploiting the mycobacterial cell wall to develop next-generation therapies capable of addressing drug resistant tuberculosis by integrating recent advances across these diverse fronts.

## 2 Targeting cell wall biosynthesis

### 2.1 Inhibition of arabinogalactan biosynthesis

Arabinogalactan is a critical component of the mycobacterial cell wall, providing structural integrity and serving as the anchor for mycolic acids. Its biosynthesis involves a series of essential enzymes, including decaprenylphosphoryl-β-D-ribose2′-oxidase(DprE1) and decaprenylphosphoryl-2-keto-β-D-erythro-pentose reductase (DprE2) for arabinose donor generation, GlfT1 and UGM for galactan chain assembly, and arabinosyltransferases such as EmbC and AftB for arabinan polymerization and terminal modification. Inhibition of these enzymes disrupts cell wall synthesis and has become a major focus of anti-tuberculosis drug development.

#### 2.1.1 Inhibitors targeting DprE1

DprE1 is a key enzyme in the arabinogalactan biosynthetic pathway, catalyzing the conversion of decaprenylphosphoryl-β-D-ribose (DPR) to decaprenylphosphoryl-β-D-arabinose (DPA), an essential step for mycobacterial cell wall assembly. Its essentiality and absence in humans have established it as a highly validated target for anti-tuberculosis drug development. Progress has been made in several directions, including the discovery of novel DprE1 inhibitors, structural optimization of existing scaffolds to improve druggability, computational strategies to overcome resistance mutations, and preclinical efficacy studies in clinically relevant animal models.

##### 2.1.1.1 Discovery of novel DprE1 inhibitors

The discovery of novel DprE1 inhibitors has become a major focus of anti-tuberculosis drug development, given that DprE1 has emerged as a highly selective and vulnerable target. Through phenotypic screening, a purine lead was optimized to analogues 56 and 64 (MIC 1 μM against H37Rv and resistant strains) (Finger et al., 2023), and the carbostyryl derivative OPC-167832 was validated as a DprE1 inhibitor with exceptionally low MICs (0.00024–0.002 μg/mL) (Hariguchi et al., 2020). Pflégr et al. synthesized N-substituted 5-(3,5-dinitrophenyl)-1,3,4-oxadiazol-2-amine derivatives that exhibited potent activity (MIC ≤0.03 μM) via DprE1 inhibition, leading to accumulation of trehalose monomycolates and dimycolates (Pflégr et al., 2025). Virtual screening identified a quinoxaline ligand with high binding affinity (−10.6 kcal/mol) that restored stability against the C387N mutant (El Haddoumi et al., 2024) and benzimidazole derivatives with compound 21 showing favorable ADMET profiles (Yalcin-Ozkat et al., 2023). However, the quinoxaline ligand has been identified solely through computational approaches and awaits experimental validation. The natural product CNP0123918 as a novel DprE1 binder with reduced toxicity (Snehalatha and Kumar, 2025). Structure-based pharmacophore modeling yielded the azaindole derivative ZINC000170252277 as a non-covalent inhibitor (Kb et al., 2020), while ChEMBL database screening identified C6 with superior binding free energy compared to CT319 (Nir et al., 2021), and hydantoin-based compounds led to ZINC12196803 as a promising non-covalent inhibitor (Mali et al., 2022). Bis-benzothiazole amides optimized from virtual screening exhibited potent activity against drug-resistant strains with binding at Tyr314 (Samoon et al., 2024), and a systematic study of morpholino-pyrimidine inhibitors revealed that MP-38 induced stable folding of the L-I and L-II loops (Tayal et al., 2025). Hybrid molecules were designed and validated to stably engage the active site with low cytotoxicity, including 1,2,3-triazole-benzoxazole (BOK-2, BOK-3) and pyrazole-1,2,4-triazole conjugates (e.g., 7j) (Singh et al., 2024; Ajin et al., 2023). In parallel, the 6-nitropicolinamide derivative 77 demonstrated high oral bioavailability (63%) and covalent binding to Cys387 (Thompson et al., 2026).

##### 2.1.1.2 Optimization of known DprE1 scaffolds

Optimizing known DprE1 scaffolds is critical for balancing ultra-high potency with favorable drug-like properties, thereby overcoming the pharmacokinetic and physicochemical limitations that hinder the clinical development of otherwise highly active anti-tuberculosis agents. Based on the benzothiazinone scaffold, Fan et al. introduced a 6-methanesulfonyl substitution and employed five-membered aromatic heterocycles as linkers to modify the aryl side chain, and found that the optimized compounds 6 and 38 exhibited improved aqueous solubility and higher metabolic stability in human liver microsomes compared to the lead compound PBTZ169, while maintaining nanomolar MIC values (47 nM and 30 nM, respectively) against Mtb (Fan et al., 2021). A scaffold hopping strategy was employed to transplant the benzenoid ring of PBTZ169 onto a quinolone nucleus, leading to the synthesis of 22 novel quinolone-benzothiazinone hybrid analogues. Among these, compound 25 retained activity against the DprE1 P116S mutant but showed reduced activity against the C387S mutant, confirming the critical role of the C387 residue in inhibitor binding (Dube et al., 2023). To overcome the poor aqueous solubility of PBTZ169, Shi et al. replaced the 6-trifluoromethyl group with a more hydrophilic methanesulfonyl moiety, leading to the design and synthesis of a series of 6-methanesulfonyl-8-nitrobenzothiazinone derivatives. Among these, MsPBTZ169 and compounds 2 and 8 exhibited MICs below 40 nM against H37Rv along with improved solubility. However, compound 2 showed no detectable plasma concentration after oral administration in mice, underscoring the inherent difficulty in simultaneously optimizing potency, solubility, and pharmacokinetic properties (Shi et al., 2022). Further side chain modification of the benzothiazinone scaffold led to compound 37, which maintained nanomolar activity while achieving significantly improved aqueous solubility and oral bioavailability (70.6%), with *in vivo* efficacy confirmed in an acute infection mouse model (Fan et al., 2023). Based on the previously identified lead G50, 45 pyrimidinetrione derivatives were designed and synthesized, and compound 42 exhibited a 5-fold increase in DprE1 inhibitory activity compared to G50 along with a high selectivity index (SI = 186.74) surpassing linezolid (Liang et al., 2025). In parallel, structure-activity relationship studies on hydantoin-based DprE1 inhibitors yielded over 80 new derivatives, with optimized compounds achieving submicromolar cellular potency, nanomolar target affinity, balanced physicochemical properties, and low human cytotoxicity, and *in vivo* activity was validated in an acute tuberculosis infection model (Balabon et al., 2020).

##### 2.1.1.3 Resistance-guided design of DprE1 inhibitors

The rational design of next-generation DprE1 inhibitors relies on the elucidation of resistance mechanisms and the application of computational approaches. El Haddoumi et al. performed a virtual screening of 111 molecules with known DprE1 activity and identified six potential lead compounds. Analysis of 10 mutations revealed five as deleterious, including C387N. Subsequent molecular dynamics simulations demonstrated that ligand 2 restored binding stability disrupted by the C387N mutation, as evidenced by a lower RMSD (0.21 nm) and improved hydrogen bonding (El Haddoumi et al., 2024). It was found that a systematic virtual screening of 754 benzothiazinone analogs as irreversible DprE1 inhibitors, incorporating covalent docking and binding free energy calculations, identified three lead compounds (PubChem-155-924-621, PubChem-127-032-794, PubChem-155-923-972) with superior binding affinities (ΔG_binding_ values of −77.2, −74.3, and −65.4 kcal/mol) compared to PBTZ169 (−49.8 kcal/mol), and molecular dynamics simulations confirmed the structural stability of the complexes over 100 ns (Ibrahim et al., 2024). Additionally, cheminformatics and artificial intelligence analysis of approximately 1300 DprE1 inhibitors revealed marked hydrophobicity and limited chemical diversity, with automatic SAR and R-group decomposition identifying key substructures responsible for activity and machine learning approaches identifying potential toxicophores including thiophene and six-membered aromatic rings (Chhabra et al., 2021). Structure-based pharmacophore modeling of azaindole derivatives, integrating a multi-layered computational platform, identified ZINC000170252277 as a non-covalent DprE1 inhibitor with favorable binding affinity and stability (Kb et al., 2020). A comprehensive computational strategy for hydantoin-based DprE1 inhibitors, combining GA-MLR, atom-based, and field-based 3D-QSAR models, yielded an AAAHR_1 pharmacophore model and identified ZINC12196803 as a lead compound with enhanced binding characteristics (docking score: −9.437 kcal/mol, MMGBSA binding free energy: −70.508 kcal/mol) (Mali et al., 2022). This compound has been identified only *in silico* and requires experimental confirmation. Systematic investigation of 55 morpholino-pyrimidine-based DprE1 inhibitors using molecular dynamics, docking, 3D-QSAR, and ADMET analysis revealed that MP-38 induced stable folding of the L-I and L-II loops while reducing protein mobility, and a robust 3D-QSAR model demonstrated that negatively charged groups near rings A and B, together with hydrophobic and bulky groups near rings D and E, significantly enhanced inhibitory activity (Tayal et al., 2025).

##### 2.1.1.4 *In vivo* efficacy and lesion penetration

The *in vivo* efficacy and intralesional pharmacokinetics of DprE1 inhibitors have been extensively evaluated in animal models recapitulating the complex pathology of human tuberculosis. In the C3HeB/FeJ mouse model of caseous necrotic pulmonary lesions, a head-to-head comparison of three clinical candidates (TBA-7371, PBTZ169, and OPC-167832) revealed that lesion penetration and retention at the cellular-caseum interface and caseous necrotic core were critical determinants of efficacy, with OPC-167832 achieving a 3-log_10_ reduction in lung colony-forming units even at low doses due to its lowest MIC, favorable tissue distribution, and sustained drug concentrations above the MIC at the primary bacterial sites (Robertson et al., 2021). In the same model, the suicide inhibitor BTZ-043 administered as monotherapy for 2 months significantly reduced bacterial burdens in the lungs and spleens. Laser capture microdissection-based pharmacokinetic analysis demonstrated that BTZ-043 penetrated cellular and necrotic lesions and maintained concentrations above the serum-shifted MIC in the caseous necrotic core at the end of the dosing interval (Ramey et al., 2023). In a guinea pig model, BTZ-043 reached high concentrations within *Mycobacterium* bovis BCG-induced granulomas and, after 4 weeks of treatment, significantly reduced bacterial burdens in the infection site, draining lymph nodes, and spleen, accompanied by fewer and less necrotic granulomas (Eckhardt et al., 2023). Additionally, in a mouse model of chronic tuberculosis, OPC-167832 monotherapy exhibited potent bactericidal activity starting at 0.625 mg/kg, and combinations with delamanid, bedaquiline, or levofloxacin, as well as three- or four-drug regimens centered on delamanid and OPC-167832, showed synergistic effects with superior bacterial burden reduction and relapse prevention compared to standard therapy (Hariguchi et al., 2020).

#### 2.1.2 Inhibitors targeting DprE2

The molecular target of the anti-tuberculosis drugs pretomanid and delamanid was identified as DprE2, a key subunit of decaprenylphosphoryl-ribose-2′-epimerase. Through gene overexpression experiments, it was demonstrated that DprE2 overexpression increased mycobacterial tolerance to these drugs by more than 20-fold. Enzymatic assays further revealed that Ddn-activated pretomanid specifically inhibited the conversion of DPR to DPA, with NAD(H) identified as an essential cofactor for drug activation, suggesting that an NAD-adduct may serve as the active metabolite form. DprE2 and DprE1 form a highly druggable enzyme complex. Bicyclic nitroimidazole drugs have achieved irreversible inhibition via NAD-adduct formation, opening avenues for DprE2-targeted inhibitor development and providing a rationale for optimizing existing clinical drugs (Abrahams et al., 2023).

#### 2.1.3 Inhibitors targeting galactan biosynthesis enzymes

Enzymes involved in galactan biosynthesis represent potential targets for anti-tuberculosis drug development. Galactofuranosyl transferase 1(GlfT1), the initiating enzyme for galactan chain biosynthesis, was systematically evaluated through the construction of knockdown and complemented strains in *Mycobacterium tuberculosis* H37Ra. Chauhan et al. systematically evaluated GlfT1, the initiating enzyme for galactan chain biosynthesis, using knockdown and complemented strains in *Mycobacterium tuberculosis* H37Ra. GlfT1 downregulation led to increased susceptibility to ethambutol, reduced biofilm formation, altered cell wall permeability, and significantly impaired bacterial survival in macrophages and in a mouse model, providing the first validation of GlfT1 as an underexplored vulnerable cell wall synthesis target (Chauhan et al., 2023). Moreover, It was found that using the known allosteric inhibitor MS208 as a lead structure, 13 pyrazole and triazole derivatives were designed and synthesized, and their inhibitory activity against UDP-galactopyranose mutase (UGM) was evaluated. The O-acylated derivative DA10 displayed a competitive inhibition mode with a Ki value of 51 ± 4 μM, superior to the lead compound, although no improvement in vitro anti-tuberculosis activity was observed, highlighting the challenge of translating potent enzyme inhibitors into cell-active antibacterial agents (Ahmed et al., 2022). In contrast, three heterocyclic structures were screened as potential UGM inhibitors using docking simulations. An “oxazepino-indole” structure was successfully identified as a novel UGM inhibitor that exhibited *in vitro* activity against Mtb growth, offering a promising molecular starting point for developing novel anti-tuberculosis drugs targeting this bacteria-specific enzyme (Maaliki et al., 2020).

#### 2.1.4 Inhibitors targeting arabinosyltransferases

Arabinosyltransferases represent important targets for disrupting the assembly of arabinogalactan and lipoarabinomannan in the mycobacterial cell wall. It was found through molecular docking studies that the binding affinities of ethambutol, isoniazid, and five ethambutol-modified derivatives with *Mycobacterium tuberculosis* arabinosyltransferase C (EmbC) were evaluated. The modified molecules Emb1 and Emb3 exhibited superior binding energies (−5.77 kcal/mol and −5.13 kcal/mol, respectively) compared to the parent compound ethambutol, indicating their potential as promising EmbC inhibitors (Das et al., 2020), although these computational predictions await experimental validation. The high-resolution cryo-EM structures of the mycobacterial glycosyltransferase AftB were determined in its apo state and in complex with the donor substrate analog 2F-FPA, and it was shown that AftB adopts a GT-CA fold with its transmembrane and periplasmic domains forming an irregular tube-shaped cavity that serves as the critical region for substrate binding and catalysis. Furthermore, through integration of biochemical experiments with molecular dynamics simulations, the catalytic residue D62 and key substrate-binding residues including R221 and R372 were identified, and a double-displacement mechanism was proposed for the formation of β-(1→2) arabinosidic linkages (Liu Y. et al., 2025). To systematically investigate the structural requirements of ethambutol, three series of novel analogs were designed and synthesized, including analogs retaining the ethylenediamine scaffold with fluorine substitution or phenyl ring modifications, as well as “reversed” analogs with exchanged nitrogen and oxygen atom positions. It was demonstrated that only analogs highly similar to ethambutol in structure, such as compound 2 with side chain ethyl groups replaced by methyl groups, exhibited comparable anti-tuberculosis activity, while fluorine substitution, phenylring modifications, or atom position exchange led to reduced or complete loss of activity, highlighting the highly conserved structural requirements of ethambutol (Abdelaziz et al., 2025).

### 2.2 Inhibition of peptidoglycan biosynthesis

Peptidoglycan is an essential component of the mycobacterial cell wall, providing structural integrity and protection against osmotic lysis. Its biosynthesis involves a complex pathway encompassing precursor synthesis in the cytoplasm, lipid-linked intermediate assembly at the membrane, and extracellular cross-linking and remodeling. Given its essentiality and the absence of many of its biosynthetic enzymes in humans, the peptidoglycan pathway has long been recognized as a fertile ground for anti-tuberculosis drug development. Recent advances have expanded the repertoire of potential targets within this pathway, ranging from early-stage enzymes (MurA, MurT-GatD, DapB, MurI) involved in precursor generation and modification, to later-stage players including L,D-transpeptidases, penicillin-binding proteins, and DacB1 that mediate cross-linking and cell wall maturation. In addition, emerging evidence has highlighted the importance of peptidoglycan hydrolases (RipA, Ami1, NlpC_P60 family) in cell division and remodeling, as well as recycling pathways (NagA) and interconnected metabolic nodes (MtPrsA) as novel vulnerabilities.

#### 2.2.1 Inhibitors of precursor synthesis and modification

Targeting the early steps of peptidoglycan precursor synthesis and modification is a critical strategy for developing novel anti-tuberculosis agents. Computer-aided drug design approaches identified four peptidomimetic compounds as potential inhibitors of MurA, the key transferase catalyzing the initial step in peptidoglycan biosynthesis. Molecular dynamics simulations confirmed their stable binding with catalytic site residues of MurA and favorable pharmacokinetic profiles (Kumar et al., 2020). It was established that D-glutamate amidation in peptidoglycan precursors is essential. Knockdown of the MurT-GatD complex via CRISPR interference was found to be lethal, causing severe growth defects, reduced peptidoglycan cross-linking, and increased sensitivity to lysozyme and β-lactam antibiotics, thereby identifying MurT-GatD as a novel and vulnerable drug target (Shaku et al., 2023). Additionally, the Rv3712 and Rv3713 genes form an operon encoding the MurT/GatD enzyme complex. Rv3712 possesses ATPase activity and forms a heterodimer with Rv3713 that interacts with cell division and regulatory proteins, further validating MurT/GatD as a novel anti-tuberculosis drug target (Maitra et al., 2021). Angrish et al. validated dihydrodipicolinate reductase (DapB) as an essential target by constructing a dapB antisense knockdown mutant, and identified a lead compound B59 through virtual screening of approximately 95,000 compounds. This compound demonstrated effective inhibition of DapB enzymatic activity (IC_50_ 11 μg/mL) and anti-mycobacterial activity (MIC_99_ 20 μg/mL) with no significant cytotoxicity (Angrish et al., 2023). The anti-tubercular potential of natural flavonoids (naringenin and quercetin) was investigated as inhibitors of glutamate racemase (MurI), and it was found that these flavonoids effectively bound to the active site of MurI, significantly inhibited its racemization activity (competitive inhibition with Ki values of 23.8 µM and 20.8 µM, respectively), and disrupted mycobacterial membrane integrity, confirming their mechanism of action through interference with peptidoglycan biosynthesis (Pawar et al., 2020).

#### 2.2.2 Inhibitors of cross-linking and remodeling

Enzymes responsible for peptidoglycan cross-linking and remodeling have also emerged as promising targets. The inhibitory activities of β-lactam antibiotics and LdtMt2 inhibitors against L,D-transpeptidases and penicillin-binding proteins were systematically evaluated using fluorescent peptide probes, and it was revealed that dual targeting of L,D-transpeptidases(Ldts) and penicillin-binding proteins(PBPs) was necessary for achieving effective anti-mycobacterial activity, with Ldt inhibition showing a stronger correlation with bacterial growth suppression (de Munnik et al., 2025). Furthermore, The systematic evaluation of β-lactam antibiotics combined with β-lactamase inhibitors demonstrated that carbapenems (imipenem, meropenem, tebipenem) form stable acyl-enzyme complexes with the D,D-carboxypeptidase DacB1, with imipenem showing the fastest binding kinetics,and that the addition of β-lactamase inhibitors further enhances efficacy (Nantongo et al., 2025). Moreover, the diazabicyclooctane durlobactam was demonstrated to significantly enhance the efficacy of β-lactam antibiotics against M. abscessus through a dual mechanism, acting as a potent inhibitor of the β-lactamase Bla_Mab_ while also directly inhibiting key peptidoglycan synthesis enzymes including L,D-transpeptidases and D,D-carboxypeptidase, with triple combinations showing exceptionally potent *in vitro* activity (Dousa et al., 2022). Durlobactam was further shown to function as a dual inhibitor in Mtb, targeting both the BlaC β-lactamase and key peptidoglycan transpeptidases (PonA1, LdtMt1, LdtMt2, LdtMt3), with biochemical assays and mass spectrometry confirming the formation of stable acyl-enzyme complexes and antibiotic susceptibility testing revealing intrinsic activity against Mtb isolates as well as significant potentiation of β-lactam efficacy (Nantongo et al., 2024). Healy et al. characterized the critical roles of two peptidoglycan hydrolases, RipA and Ami1, revealing that RipA serves as the major hydrolase for cell division. Its depletion led to elongated multiseptated chains and increased susceptibility to cell wall-targeting antibiotics, while Ami1 was found to be crucial for persistence during chronic infection, demonstrating partial functional redundancy yet distinct roles at different stages of infection (Healy et al., 2020). It was comprehensively reviewed that the NlpC_P60 peptidase family in mycobacteria comprises five domain-containing proteins (RipA, RipB, RipC, RipD, and RipE), with RipA and RipB playing critical but non-redundant roles in cell division, RipC involved in maintaining cell wall integrity, and RipE associated with biofilm formation and drug resistance, representing underexplored anti-mycobacterial drug targets (Do et al., 2025).

#### 2.2.3 Inhibitors of recycling and metabolism

Peptidoglycan recycling and associated metabolic pathways have emerged as novel intervention points. A NagA gene deletion mutant was analyzed with multiple complementary approaches, which identified NagA as the sole enzyme responsible for the deacetylation of N-acetylglucosamine-6-phosphate in mycobacteria. Loss of NagA led to altered peptidoglycan cross-linking, thickened cell wall, and significantly increased susceptibility to multiple cell wall-targeting agents, revealing the NagA-mediated peptidoglycan recycling pathway as a potential drug target (Guy et al., 2025). Furthermore, three phytocompounds (hesperidin, rebaudioside A, and rutin) were identified as novel inhibitors of Mtb phosphoribosyl pyrophosphate synthetase (MtPrsA) through systematic screening, molecular docking, and molecular dynamics simulations. Enzyme inhibition assays confirmed their inhibitory activity with distinct mechanisms, establishing MtPrsA as a viable drug target (Manthattil Vysyan et al., 2024).

### 2.3 Inhibition of mycolic acid biosynthesis

Mycolic acids are long-chain fatty acids that form an essential component of the mycobacterial cell envelope, contributing to its impermeability and virulence. Their biosynthesis involves a complex pathway encompassing the fatty acid synthase I(FAS-I) system for precursor synthesis, the fatty acid synthase II(FAS-II) system for chain elongation, and numerous modifying and transporting enzymes. Given their essentiality and uniqueness to mycobacteria, enzymes within this pathway have long been validated targets for anti-tuberculosis therapy. Recent advances have expanded the repertoire of inhibitors targeting these enzymes, ranging from the Ag85 complex and InhA to MabA, KasA, Pks13, and FadD32, among others.

#### 2.3.1 Inhibitors targeting the Ag85 complex

The Ag85 complex, comprising Ag85A, Ag85B, and Ag85C, catalyzes the transfer of mycolic acids to the cell wall, representing a critical late-stage step in mycolic acid assembly. Amino-pyrazoline derivatives were discovered as a novel class of dual-function anti-tuberculosis agents, and lead compounds AP-02 and AP-05 were optimized to show enhanced efficacy against mycobacteria, with mechanistic studies identifying Ag85C as their primary target, thereby disrupting late-stage mycolic acid biosynthesis and impairing cell wall integrity (Cui et al., 2025). A series of oxadiazolone-core derivatives were evaluated for antibacterial activity against *Mycobacterium* abscessus, with iBpPPOX identified as the most potent compound. Activity-based protein profiling combined with mass spectrometry revealed that iBpPPOX forms a covalent adduct with the catalytic serine residue of Ag85C, identifying it as a primary vulnerable target (Madani et al., 2020). The structural dynamics of Ag85C following covalent inhibition by the β-isomer monocyclic enolphosphorus compound Cycliphostin (CyC8β) were investigated by bioinformatics and molecular dynamics simulations. Covalent binding to the catalytic serine 124 residue induced significant structural instability and flexibility in Ag85C, particularly in key loops L1 and L2, thereby identifying potential hot spots for structure-based design of novel irreversible inhibitors (Adewumi et al., 2022).

#### 2.3.2 Inhibitors targeting InhA

InhA (enoyl-acyl carrier protein reductase) is a key enzyme in the FAS-II elongation cycle and the well-established target of isoniazid. Several prodrug-type inhibitors requiring KatG activation have been reported. Mutual esters of 2-(2-isonicotinoylhydrazineylidene)propanoic acid and twenty related propanamide analogs showed excellent activity against drug-sensitive and multidrug-resistant Mtb via InhA targeting (Pflégr et al., 2021). Four pyridine carboxamide analogs from phenotypic screening also depended on KatG activation, with resistance to two linked to novel katG mutations that do not confer cross-resistance to isoniazid (Chengalroyen et al., 2020). Direct InhA inhibitors have been developed through various design strategies. Mubarak et al. demonstrated that diaryl ether dehydrozingerone derivatives (7, 14) exhibited bactericidal activity (MBC 4 and 8 μg/mL) and bound to wild-type and mutant InhA, with activity against isoniazid-resistant and MDR isolates (Mubarak et al., 2025). A 1,5-triazole bis-triclosan derivative (2) showed IC_50_ 5.6 µM against InhA, and compound 11 gave MIC_99_ 12.9 µM in M. bovis whole-cell assays (Armstrong et al., 2020) 1,2,4-triazole-based compounds (6h,6i,6l,11c) had IC_50_ 1.3–4.7 µM and activity against MDR/XDR strains (Zawal et al., 2023). A diphenyl ether-pyrazoline hybrid (PYN-8) showed MIC 4–7 μM with a Tyr158 hydrogen bond and π-π stacking with NAD^+^ (Tiwari et al., 2020). Computational approaches identified further leads. An integrated machine-learning and pharmacophore screen yielded JFD01724 (Kuldeep et al., 2021). A cheminformatics screen of the ASINEX database gave B244, B369, and B310 as potential inhibitors of isoniazid-resistant strains (Kamera et al., 2024). A 3D-QSAR/pharmacophore model based on 47 compounds led to pyrazole derivatives (5a,5c,5d,5e) with MICs 2.23–4.61 µM (Modi et al., 2023). SAR optimization of the KES4 A-ring produced two improved candidates (3d,3f) (Taira et al., 2020). Finally, in a nutrient starvation model, all tested InhA inhibitors (including diazaborine derivatives AN12855, AN12541) reduced viability of non-replicating Mtb by more than 3 logs over 21 days, confirming that InhA is an effective target even in non-replicating populations (Flint et al., 2020).

#### 2.3.3 Inhibitors targeting other FAS-II enzymes

Beyond InhA, other enzymes within the FAS-II elongation cycle represent additional drug targets. A large-scale virtual screening of 1,792,771 compounds against MabA (FabG1) identified 48 novel lead compounds from five distinct classes, with 47 compounds showing MM/PBSA binding free energies significantly higher than the only previously reported MabA inhibitor (Panda et al., 2026). All these compounds have been identified only *in silico* and no experimental validation has been reported. Fragment-based screening using a novel LC-MS/MS assay discovered the first specific small-molecule MabA inhibitors. An anthranilic acid fragment was optimized into more potent inhibitors, and binding was confirmed by ^19^F ligand-observed NMR (Faïon et al., 2020). Structure-based virtual screening against KasA identified six top-ranking compounds that bound strongly to the malonyl site, with computed binding free energies significantly lower than the reference KasA inhibitor TLM5 (Andrianov et al., 2025). These computational predictions require experimental confirmation. A computational screen of 730 molluscan metabolites using docking, ADMET prediction, and molecular dynamics identified four compounds (CMNPD7125, CMNPD22991, CMNPD4542, and CMNPD12265) as potential KasA inhibitors, which exhibited favorable ADMET profiles and stable protein-ligand binding over 200 ns simulations (Alanzi et al., 2025), but these are purely computational predictions without experimental validation. KEGG pathway analysis identified mtFabH as a key regulatory enzyme. Structure-based virtual screening of the ChEMBL library yielded two lead compounds, ChEMBL414848 (C1) and ChEMBL363794 (C2), with superior docking scores to thiolactomycin, and molecular dynamics showed that C2 had the lowest binding free energy (Kumar et al., 2022). Structure-based virtual screening of the NPASS natural product library identified three novel FabD inhibitors, among which NPC313985 exhibited the most stable interaction with FabD in molecular dynamics simulations, thereby disrupting mycolic acid biosynthesis (Sundararajan et al., 2024).

#### 2.3.4 Inhibitors targeting Pks13

Polyketide synthase 13 (Pks13) catalyzes the final condensation step in mycolic acid biosynthesis, an essential process for mycobacterial cell wall assembly. Trehalose-Pks13 inhibitor conjugates constructed via click chemistry demonstrated that trehalose modification enhanced anti-mycobacterial potency, validating the “Trojan Horse” strategy for overcoming cell wall permeability (Kumbalathara et al., 2025). The covalent compound CMX410 irreversibly targets the acyltransferase domain of Pks13 through a β-lactam, showing high potency against clinical isolates and oral bioavailability (Krieger et al., 2025). BMVC-8C3O was found to inhibit Pks13 via binding confirmed by surface plasmon resonance and molecular docking. Mutations of Asn1640 and Ser1533 reduced its binding affinity (Liu T. et al., 2025). Phenotypic screening identified a pentafluorophenyl warhead lead that targets Pks13. Optimization yielded compound 43 with improved metabolic stability while retaining activity, confirming Pks13 as a drug target (Green et al., 2025). Coumestan derivatives were identified as a novel class of Pks13 inhibitors. Lead compound 1 showed potent bactericidal activity, dose-dependent efficacy in mice, synergy with rifampin, and favorable bioavailability (Lun et al., 2023). A scaffold hopping strategy produced 2,4,5-substituted benzoxazole derivatives with potent *in vitro* activity, low cytotoxicity, and on-target effects mapped to the Pks13 thioesterase domain (Obadawo et al., 2025). An integrated computational screen of the Asinex library identified three leads (BBB_26582140, BBD_30878599, BBC_29956160) targeting the Pks13 thioesterase domain, with 300 ns MD simulations confirming stable complex formation (RMSD ≤3 Å) and favorable pharmacokinetic profiles (Altharawi et al., 2023).

#### 2.3.5 Inhibitors targeting other mycolic acid-related enzymes

Several other enzymes involved in mycolic acid modification and activation have also been targeted. Compounds 6c and 6i from a series of 2-[(2-amino-6-methylpyrimidin-4-yl)sulfanyl]-N-arylacetamide derivatives exhibited anti-tuberculosis activity threefold more potent than ethambutol, with docking studies suggesting selective binding to cyclopropane mycolic acid synthase 1 (CmaA1) (Erkin et al., 2023). Mtb epoxide hydrolase EphD was confirmed as a target of urea and thiourea derivatives. The isoniazid analog isoxyl and the MmpL3 inhibitor AU1235 significantly inhibited EphD activity, and mycolic acid profile analysis confirmed inhibition within bacterial cells (Madacki et al., 2021). Nitroimidazole oxazine derivatives targeting the deazaflavin-dependent nitroreductase (Ddn) were analyzed using 3D-QSAR, docking, and molecular dynamics, yielding CoMFA and CoMSIA models with good predictive ability. Key residues (Tyr65, Ser78, Tyr130, Tyr133, Tyr136) contributed significantly to binding energy, guiding rational design (Yang et al., 2025). The isoxazole derivative M1 specifically inhibited fatty acyl-AMP ligases FadD32 and FadD28, regulated cell wall synthesis genes, and reduced bacterial burden and granulomas in infected macrophages and a mouse chronic infection model (Rani et al., 2025). High-throughput screening of the Prestwick Chemical Library identified the salicylanilide closantel as a potent FadD32 inhibitor, with direct binding confirmed by thermal shift assays and docking, IC_50_ 7.7 µM, and MIC 0.08 µM against Mtb (Le et al., 2022). Whole-cell phenotypic screening identified azetidine derivatives (BGAz) that disrupt cell envelope biogenesis by targeting late stages of mycolic acid biosynthesis through a mechanism distinct from existing inhibitors (Cui et al., 2024). A 1,3-diarylpyrazolyl-acylsulfonamide hit from phenotypic screening in cholesterol-containing medium exerted bactericidal activity by disrupting cell wall biosynthesis via a novel mechanism distinct from MmpL3, DprE1, InhA, and EthA (Khonde et al., 2021).

#### 2.3.6 Inhibitors targeting MmpL3

MmpL3 is a mycolic acid transporter that plays a critical role in the translocation of trehalose monomycolates across the inner membrane.

##### 2.3.6.1 Discovery and optimization of MmpL3 inhibitors

Several screening campaigns have identified diverse chemical scaffolds targeting MmpL3. A focused library of 400 synthetic compounds yielded novel phenyl urea derivatives with sub-micromolar activity. Chemical proteomics identified MmpL3 along with EphD and EphF as targets, and structural optimization produced a lead with enhanced activity, low cytotoxicity, improved solubility and oral bioavailability (Mostert et al., 2025). Among dihydrosphingosine and ethambutol analogs, 11 compounds showed good activity. Lead 12b was confirmed to target MmpL3, exhibiting far greater potency than ethambutol, with killing that is dependent on time and concentration, and synergy with rifampicin (Linhares et al., 2023). Re-evaluation of 2,5,6-trisubstituted benzimidazoles by bacterial cytological profiling revealed cell wall damage and ATP accumulation, clarifying that these compounds target MmpL3 rather than cell division (Zhang M. et al., 2025). Thienopyrimidine amide analogs were also confirmed to target MmpL3, inducing cell wall stress and characteristic ATP elevation (Baldin et al., 2025).

Computational approaches have further accelerated inhibitor discovery and optimization. Structure-based high-throughput virtual screening combined with molecular dynamics identified four inhibitors (C1, C3, C7, C9) from approximately 17 million compounds. These compounds occupy the proton channel and disrupt key aspartate-tyrosine pairs (D256-Y646 and D645-Y257), thereby blocking mycolic acid precursor transport (Choksi et al., 2024). A pharmacophore-based computational workflow employing SQ109 as a lead yielded two candidates (ZINC000000016638, ZINC000000003594), with the former showing superior stability in MD simulations (C et al., 2023). Fifty pyrazole amide derivatives were designed and synthesized. Compounds 15 and 35 exhibited potent activity against drug-sensitive and resistant strains, and docking, molecular dynamics, and genetic analysis of SQ109-resistant strains confirmed MmpL3 targeting (Maddipatla et al., 2025). Fifty adamantane and adamantanol indole-2-carboxamides were also prepared. Compounds 8j and 8k showed excellent activity, with docking revealing a binding mode similar to ICA38 and AU1235, occupying the S3-S5 subsites and disrupting Asp-Tyr proton relay pairs (Alsayed et al., 2021). The arylsulfonamide TPN-0157345 was shown to target MmpL3 via bacterial cytological profiling (morphological changes similar to AU1235), iniB induction, ATP elevation, and low-level resistance in a strain carrying the S591I mutation (Allen et al., 2024). In addition to directly targeting MmpL3, the inhibitor SQ109 has been reported to exert its bactericidal effect primarily through respiration inhibition. Using the computational tool DECIPHAER, Johnson et al. revealed that SQ109 kills *M. tuberculosis* mainly by disrupting energy metabolism rather than its previously known cell-wall effects, a finding confirmed by ATP assays and cell survival experiments. Thus, SQ109 may have a dual mechanism of action involving both MmpL3 inhibition and respiratory chain disruption (Johnson et al., 2025). Collectively, these studies have greatly expanded the chemical diversity of MmpL3 inhibitors and provided optimized leads with improved drug-like properties.

##### 2.3.6.2 Structural insights into MmpL3 inhibition

A structure-based design approach was employed to develop a series of novel compounds targeting MmpL3, and the lead molecule ST004 exhibited potent inhibitory activity against Mtb in cell culture, with cryo-electron microscopy resolving the complex structure of ST004 bound to MmpL3 within lipid nanodiscs, revealing that ST004 binds to the inhibitor-binding pocket within the proton translocation channel with critical roles played by the S4 and S5 subsites (Hu et al., 2022).

##### 2.3.6.3 Resistance mechanisms and pharmacological characterization

**FIGURE 2:**
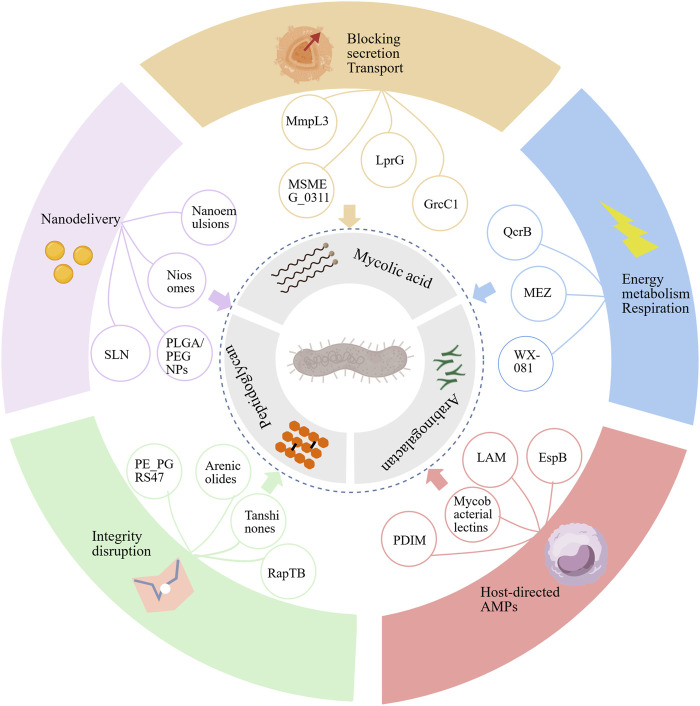
Multidimensional modulation of the mycobacterial cell wall. The inner ring summarizes traditional strategies targeting biosynthetic enzymes of mycolic acids, arabinogalactan, and peptidoglycan. The outer ring highlights five emerging approaches with their representative targets/molecules: disruption of cell wall integrity (green), energy metabolism and respiration (blue), blocking secretion and transport (orange), host-directed therapy and AMPs (red), and nanotechnology-based delivery (purple).

The S288T mutation in MmpL3 was investigated using molecular dynamics and quantum mechanics simulations, revealing that it induces local conformational changes in the periplasmic channel, rotates residue Y44, and causes pronounced channel bending, thereby reducing substrate transport efficiency and conferring resistance to SQ109 (Ge et al., 2024). Sequential selection of resistant mutants against structurally diverse MmpL3 inhibitors showed that all resistant isolates carried mmpL3 mutations, with multiple substitutions (e.g., F255L, L567P, V646M, F644I/L) accumulating under increasing pressure, leading to higher resistance levels and broader cross-resistance without a fitness cost (McNeil et al., 2020). A systematic comparison of eleven MmpL3 inhibitor series revealed common microbiological properties, including potent activity against replicating Mtb, enhanced efficacy against intramacrophage bacilli, rapid bactericidal action, and cell wall stress accompanied by increased ATP levels, while being inactive against dormant bacilli (Ames et al., 2025). Berube et al. investigated the kill kinetics of NITD-349 and found significant inoculum dependence, achieving rapid sterilization at low bacterial densities but reduced kill rates at high densities. Combination with isoniazid synergistically enhanced killing and prevented the emergence of resistant mutants (Berube et al., 2023). Overall, these findings delineate the resistance landscape and pharmacological profile of MmpL3 inhibitors, thereby guiding strategies to circumvent resistance and optimize treatment regimens. As shown in Figure 2 (Jiang et al., 2025), a conceptual framework integrates conventional and innovative cell wall-targeting strategies, including disruption of integrity, energy metabolism regulation, blocking secretion, host-directed therapy, and nanodelivery.

## 3 Disruption of cell wall integrity

Diverse mechanisms have been implicated in the disruption of mycobacterial cell wall integrity. Knoll et al. employed a GC × GC-TOFMS metabolomics approach and revealed that the decoquinate derivative RMB041 caused significant metabolic alterations in fatty acid, amino acid, glycerol metabolism, and the urea cycle, with accumulation of fatty acids (e.g., C10:0–C20:0, Δ^9^ C16:1, Δ^9^ C18:1) and precursors such as glycerol-3-phosphate and myo-inositol, indicating that RMB041 primarily disrupts mycobacterial cell wall biosynthesis (Knoll et al., 2021). Among polycyclic amine derivatives, compounds 14 and 15 exhibited strong activity against Mtb H37Rv, and a PiniB-LUX reporter assay showed they caused early and sustained cell wall damage similar to ethambutol (Kapp et al., 2021). Heterologous expression of the PE_PGRS47 protein in *Mycobacterium* smegmatis altered colony morphology, cell wall lipid composition, and increased permeability, leading to enhanced susceptibility to multiple antibiotics and stressors (Li et al., 2023). Eight new arenicolide macrolides (Ar-D to Ar-K) were isolated from Micromonospora sp. GR10. Ar-A displayed potent bactericidal activity against MDR/XDR Mtb through ATP depletion and cell wall disruption, reduced bacterial burden and pulmonary pathology in zebrafish and mouse models, and showed efficacy against intracellular bacteria (Hwang et al., 2025). It was demonstrated that three tanshinones (tanshinone I, IIA, cryptotanshinone) from Salvia miltiorrhiza exhibited MIC_90_ values of 0.38–1.21 μg/mL against drug-resistant strains with low cytotoxicity, while scanning electron microscopy revealed morphological cell wall changes and resistant mutants harbored mutations in PE_PGRS genes, suggesting the cell wall as a target (Polinário et al., 2025). A 39-amino acid peptide (RapTB, from hemoglobin β chain HBB 112-147) identified from a human lung peptide library exerted bactericidal activity against extracellular Mtb by disrupting cell wall integrity, with low toxicity to primary human macrophages (Klevesath et al., 2025). Thus, disrupting cell wall integrity via multiple mechanisms offers a promising strategy to overcome drug-resistant tuberculosis.

## 4 Metabolic and energy regulation of cell wall synthesis

Mycobacterial cell wall synthesis is tightly coupled to energy metabolism and regulatory networks, rendering these interconnected pathways attractive targets for anti-tuberculosis drug development.

### 4.1 Energy metabolism and respiration

The energy metabolism and respiratory chain of Mtb are essential for bacterial survival and represent critical vulnerabilities for therapeutic intervention. The crystal structure of mycobacterial malic enzyme (MEZ) was resolved, revealing that MEZ exists as a flexible dimer capable of alternating between open and closed conformations. This structural plasticity provides a foundation for designing selective inhibitors that exploit differences between MEZ and human malic enzymes, thus linking central carbon metabolism to redox balance and energy supply (Burley et al., 2021). Targeting the respiratory chain, the novel candidate Sudapyridine (WX-081), a pyridine analog of bedaquiline, showed comparable *in vitro* efficacy against drug-sensitive and drug-resistant Mtb while exhibiting weaker hERG channel inhibition and no QTc prolongation in beagle dogs, indicating reduced cardiotoxicity (Yao et al., 2022). Another key agent, The mycobacterial cytochrome b subunit (QcrB) within the cytochrome bc1 complex was inhibited by another key agent, Telacebec (Q203), thereby disrupting menaquinol-driven electron transport and ATP production (Malík et al., 2021). Furthermore, the biosynthesis of virulence lipids PDIM and PGL was downregulated by Q203, thereby sensitizing Mtb to other antibiotics such as vancomycin and rifampicin (Zhou et al., 2023). Similarly, a series of imidazo[1,2-a]pyridinecarboxamide derivatives (compounds 15 and 16) were designed to target QcrB, leading to inhibition of electron transport and proton translocation, and they maintained potent activity against multidrug-resistant and extensively drug-resistant clinical isolates (Onajole et al., 2020). Collectively, these findings underscore that disrupting energy metabolism and respiration, either through direct enzyme inhibition or by targeting the electron transport chain, constitutes a highly effective strategy against drug-resistant tuberculosis.

### 4.2 Regulation and signaling

Signaling and regulatory networks governing cell division, metabolism, and cell wall homeostasis are key anti-tuberculosis targets. A multi-level computational screen of three large libraries identified potential inhibitors of Mtb serine/threonine protein kinase B (PknB), yielding six lead compounds including Riboflavin Monophosphate and Tedizolid Phosphate. One compound (AB-00011214) induced a shift of the active site from a closed to an open conformation (Vieira et al., 2021). The transcription factor BlaI (Rv1846c) was found to regulate cell division and β-lactam susceptibility. Its overexpression delayed growth, caused cell elongation and multi-septum formation, altered cell wall lipid permeability, and increased sensitivity to β-lactams by directly repressing the key division gene ftsQ (Xu et al., 2025). Deletion of the surface adhesins MTP or HBHA altered expression of genes involved in the tricarboxylic acid cycle, oxidative phosphorylation, and cell wall transport. ATP synthase genes were upregulated but intracellular ATP levels dropped, suggesting impaired proton motive force or increased ATP consumption (Naidoo et al., 2025). Finally, the allosteric regulation of the cell wall hydrolase RipA was elucidated: the activator protein SteB forms a homodimer that engages the RipA coiled-coil domain, while the cytoplasmic domain of SteA may sense cell wall metabolic status via phosphonucleotide binding, thereby transducing regulatory signals (Carloni et al., 2025). Together, these findings highlight that targeting signaling kinases, transcription factors, adhesins, and allosteric regulators can disrupt the coordinated networks governing mycobacterial cell wall synthesis and division.

### 4.3 Multi-mechanism and computational approaches

Integrated computational and experimental strategies have uncovered diverse mechanisms of action and enabled rational inhibitor design. A synthetic-bioinformatic approach predicted structures from 96 nonribosomal peptide synthetase gene clusters and chemically synthesized 157 cyclic peptides, leading to nine new antibiotics with varied modes of action, including inhibition of cell wall biosynthesis (SyCPA 4) and disruption of membrane integrity (SyCPA 12/102/123) (Chu et al., 2020). Furthermore, *ab initio* fragment molecular orbital calculations elucidated key interactions of a benzimidazole derivative (compound 7) with InhA, showing hydrogen bonds and electrostatic contacts with Gln100 and Ala157. Structure-based design of 24 analogues identified N2-OH (with a hydroxyl at the R2 position), which formed new hydrogen bonds and electrostatic interactions with Ile215 and NAD^+^, yielding the highest total interaction energy (−118.2 kcal/mol) (Pornprom et al., 2025). These examples illustrate how multi-mechanism discovery and computational approaches synergistically advance anti-tuberculosis drug development.

## 5 Other cell wall-associated transport and secretion proteins

The transport of mycolic acids and other cell wall components across the mycobacterial cell envelope is essential for cell wall assembly and integrity. Targeting the proteins that mediate these processes has emerged as a highly effective strategy for anti-tuberculosis drug development. Additional proteins involved in lipid transport and cell wall assembly have also been explored as drug targets. Computational virtual screening identified a series of dimethylaminophenyl hydrazide small molecules that bind to the mycobacterial lipid transport protein LprG with moderate micromolar affinity, as confirmed by a fluorescent competitive binding assay. These compounds inhibit mycobacterial growth in an LprG-dependent manner, and a mutation (F123) in the LprG binding cavity conferred resistance to the potent compound LB04, thereby validating LprG as a druggable target (Bai et al., 2020). A novel resistance mechanism mediated by the previously uncharacterized polyprenyl diphosphate synthase GrcC1 was uncovered, wherein a specific A132V mutation in GrcC1 conferred low-level resistance to a broad spectrum of drugs across multiple mycobacterial species. Functional studies showed that GrcC1 overexpression decreased drug susceptibility and reduced cell wall permeability, whereas gene silencing or knockout increased permeability and drug sensitivity, establishing GrcC1 as a critical modulator of the cell wall barrier and a potential target for reversing intrinsic resistance (Fang et al., 2025). Additionally, CRISPR-Cas12-based silencing of the highly conserved MSMEG_0311 gene caused severe growth defects and altered colony morphology. The protein product localizes preferentially to the cell pole and influences polar growth. Permeability assays, drug susceptibility tests, and transcriptomic analysis confirmed its cell wall-associated function, particularly affecting expression of iniA and the sigF regulon. Thus, MSMEG_0311 is proposed as a novel drug target (Sodani et al., 2024). Thus, targeting alternative transport and secretion proteins expands the arsenal of anti-tuberculosis agents.

## 6 Other mechanisms and emerging strategies

In addition to the major cell wall biosynthesis pathways, several other targets and strategies have emerged that offer novel approaches for anti-tuberculosis drug development. These include regulatory networks controlling cell wall synthesis, unconventional enzymes involved in cell wall assembly, immunomodulatory approaches, and drug delivery strategies.

### 6.1 Regulation of cell wall synthesis and division

Transcriptional and allosteric regulators critically control cell wall synthesis and division in Mtb. The transcription factor BlaI (Rv1846c) was found to regulate cell division and β-lactam susceptibility. Its overexpression delayed growth, caused cell elongation and multi-septum formation, altered cell wall lipid permeability, and increased sensitivity to β-lactams by directly repressing the key division gene ftsQ (Xu et al., 2025). The allosteric regulation of the cell wall hydrolase RipA was elucidated through structural and biochemical studies. The activator protein SteB forms a homodimer and engages the RipA coiled-coil domain in an induced-fit manner, while the cytoplasmic domain of SteA may function as a phosphonucleotide-binding protein that senses cell wall metabolic status and transduces regulatory signals (Carloni et al., 2025). Moreover, the LytR_C domain-containing protein VirR participates in linking peptidoglycan to arabinogalactan via interaction with LCP family proteins (e.g., Lcp1). Deletion of virR led to structural cell wall abnormalities and enhanced extracellular vesicle production, revealing a link between cell wall remodeling and vesicle biogenesis (Salgueiro-Toledo et al., 2025). Overall, the coordinated networks governing mycobacterial cell wall synthesis and division can be disrupted by targeting transcription factors, hydrolase regulators, and cell wall-linkage proteins.

### 6.2 Novel targets in cell wall synthesis

Several unconventional targets beyond the classical pathways have been identified. A virtual screening of the Super Natural-II database against RmlD identified ligand-567 as a potential inhibitor, which forms hydrogen bonds with key active site residues (Asp-105, Val-158, Thr-160, Gly-161, Arg-224, Arg-256) (Ravichandran et al., 2022). The aromatic diterpenoid ebractenoid F from Euphorbia ebracteolata was found to inhibit the acetyltransferase activity of the bifunctional enzyme GlmU by competitively blocking acetyl-CoA binding and interacting with residue Ala434, exhibiting an MIC of 12.5 mg/mL against Mtb H37Ra and synergy with isoniazid (Han et al., 2022). The TB drug candidate SQ109 and its analogues were shown to act as uncouplers of the proton motive force. More importantly, cell wall biosynthesis was directly inhibited by these compounds via targeting the recycling of the essential lipid carrier decaprenyl phosphate, as confirmed by the observation that exogenous undecaprenyl phosphate rescued bacterial growth (Malwal et al., 2023). Finally, Huang et al. demonstrated that compound ACA (3-azidothiophene-2-carboxylic acid) killed Mtb by binding to the cell wall core assembly proteins CpsA1 and CpsA2, thereby inhibiting their pyrophosphatase activity and blocking arabinogalactan-peptidoglycan ligation. Resistance was mapped to a V221E mutation in the cpsA2 gene (Hu et al., 2025). Thus, the druggable target landscape within mycobacterial cell wall synthesis has been substantially broadened.

### 6.3 Chemical synthesis and immunomodulatory strategies

Chemical synthesis of cell wall glycans not only enables structural studies but also provides defined immunomodulatory agents that can fine-tune host immune responses against *Mycobacterium tuberculosis*. An orthogonal one-pot glycosylation strategy based on glycosylortho-(1-phenylvinyl) benzoates was developed, enabling the efficient chemical synthesis of the complex arabinogalactan 92-mer and its fragments (14-mer, 30-mer, 50-mer) from the Mtb cell wall. It was revealed by conformational analysis using molecular dynamics simulations and NMR spectroscopy, along with immunological studies in human cell models, that the surface-exposed 30-mer epitope induced only modest NF-κB activation while preserving cell viability (Ma et al., 2025). In a separate approach, rabbits were immunized with Mtb H37Rv cell wall components. Novel polyclonal antibodies against lipoarabinomannan (LAM), ESX-1 secretion-associated protein B (EspB), and Mtb8 (Rv0379) were purified by affinity chromatography, and ELISA, Western blot, and flow cytometry confirmed high antigen specificity and sensitivity for all three antibodies (Yan et al., 2025). Bioinformatics analyses of 11 mycobacterial lectins revealed that these proteins and their functional partners are predominantly extracellular and belong to the PE/PPE family. Molecular docking showed that quinoxalinone derivatives bind with good affinity to the C-type lectin Rv2075c, while fucoidan derivatives bind to the filamentous hemagglutinin-like lectin Rv1917c, suggesting their potential as vaccine candidates or drug targets (Sundar et al., 2021). Moreover, the mycobacterial virulence lipid phthiocerol dimycocerosate (PDIM) was found to induce upregulation of host macrophage Galectin-3 via Toll-like receptor 2 and non-canonical TGF-β pathways. Elevated Galectin-3 impaired NF-κB turnover and nuclear translocation, thereby suppressing proinflammatory cytokine profiles and facilitating bacterial immune evasion (Wang et al., 2025). Collectively, these chemical and immunomodulatory strategies expand the toolbox for targeting Mtb beyond direct enzyme inhibition.

### 6.4 Novel compounds with uncharacterized mechanisms

Several newly identified anti-tuberculosis agents exhibit promising activity, though their precise mechanisms remain incompletely defined. A series of pyrimidine-1,3,4-oxadiazole hybrids and their precursor amides were designed via molecular hybridization and isosteric replacement. The oxadiazole derivatives bearing C8–C12 alkyl chains displayed potent antimycobacterial activity (effective from 2 µM against Mtb H37Rv), with preliminary evidence linking their action to inhibition of cell wall biosynthesis (Pflég et al., 2023). Another novel candidate, Sudapyridine (WX-081), a pyridine analog of bedaquiline, was developed to improve safety. Preclinical studies showed that WX-081 had comparable *in vitro* efficacy against drug-sensitive and resistant Mtb while exhibiting weaker hERG channel inhibition and no QTc prolongation in beagle dogs, indicating a substantially reduced cardiotoxicity risk (Yao et al., 2022). Although WX-081 is known to target ATP synthase, its full mechanism of action and the basis for its improved cardiac profile are still under investigation, placing it among compounds with not-fully-elucidated mechanisms. These findings highlight the value of exploring novel chemical entities with distinct or yet-to-be-clarified modes of action.

## 7 Drug delivery systems for enhanced cell wall penetration

The complex and highly hydrophobic cell wall of *Mycobacterium tuberculosis* poses a major barrier to conventional anti-tubercular drugs, limiting their intracellular accumulation and contributing to poor treatment outcomes and drug resistance. Nanocarrier-based drug delivery systems offer a versatile platform to overcome this barrier by improving drug solubility, protecting the payload from premature degradation, enabling targeted delivery to infected macrophages, and providing sustained or triggered release. Moreover, certain nanomaterials possess intrinsic antimicrobial or immunomodulatory activities that synergize with encapsulated drugs.

### 7.1 Nanocarriers for enhanced cell wall penetration

**FIGURE 3:**
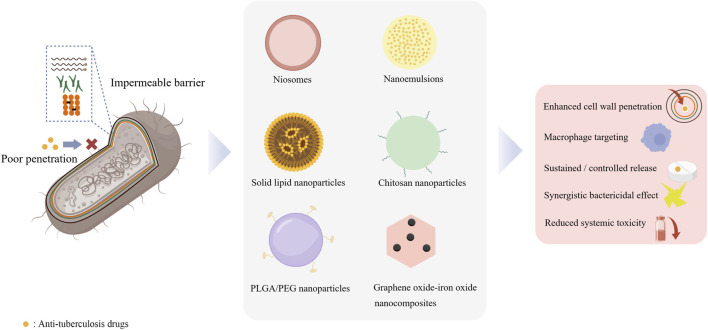
Novel drug delivery systems for enhanced cell wall penetration. The left panel shows the impermeable mycobacterial cell wall with a red cross mark indicating poor drug penetration. The center panel displays six representative nanocarriers: niosomes, solid lipid nanoparticles, PLGA/PEG nanoparticles, essential oil-based nanoemulsions, chitosan nanoparticles, and graphene oxide-iron oxide nanocomposites. The right panel lists five therapeutic benefits: enhanced cell wall penetration, macrophage targeting, sustained/controlled release, synergistic bactericidal effect, and reduced systemic toxicity. Orange circles represent drug molecules.

Various nanocarriers have been designed to improve the physicochemical properties and cellular uptake of anti-tubercular drugs. Solid lipid nanoparticles co-loaded with rifampicin, isoniazid and pyrazinamide (SLN8) achieved entrapment efficiencies above 81%, sustained release, and two-fold higher activity against *Mycobacterium* marinum in macrophages compared to free drugs (Khatak et al., 2020). Isoniazid-loaded solid lipid nanoparticles for ocular delivery extended release to 48 h, enhanced corneal permeability 1.6-fold, and improved ocular bioavailability 4.2-fold (Singh et al., 2019). Ethambutol-loaded solid lipid nanoparticles were converted into dry powder inhalers with suitable aerodynamic properties and good biocompatibility (Nemati et al., 2019). Niosomes encapsulating ethambutol hydrochloride exhibited biphasic controlled release and improved lung targeting (El-Ridy et al., 2015). A novel isatin-isonicotinohydrazide hybrid (WF-208) incorporated into niosomes showed a 4-fold increase in anti-mycobacterial activity against H37Rv compared to the free compound, with enhanced stability under simulated gastric conditions (Eldehna et al., 2022). Dihydroartemisinin-loaded chitosan nanoparticles disrupted the cell wall of rifampicin-resistant Mtb as confirmed by electron microscopy and metabolomics (Gu et al., 2021). Rifampicin-loaded PLGA nanoparticles assembled into porous nanoparticle-aggregate particles for dry powder inhalation. After intratracheal administration, rifampicin remained detectable in lung tissue for 8 h and achieved a bioavailability of 0.72, markedly higher than oral administration (Sung et al., 2009). A microemulsion co-encapsulating rifampicin, isoniazid and pyrazinamide resolved the stability issue of rifampicin in the presence of isoniazid and provided differential release profiles (Kaur et al., 2015). Essential oil-based nanoemulsions (clove oil, cinnamon oil, eugenol) loaded with four first-line drugs exhibited sustained release for over 10 h and minimum inhibitory concentrations below 0.0009 mg/mL, outperforming free drugs (Menon et al., 2023). PLGA-PEG nanoparticles functionalized with a transferrin receptor-binding peptide delivered clofazimine across the blood-brain barrier for tuberculous meningitis, reducing cytotoxicity and enhancing transcellular permeability in brain endothelial cells (de Castro et al., 2021). Ethambutol loaded on graphene oxide-iron oxide nanocomposites provided sustained release for 50 h and superparamagnetic properties for magnetic targeting (Saifullah et al., 2017). As shown in Figure 3 (Jiang et al., 2025), different nanocarriers improve drug penetration, enable macrophage targeting, prolong half-life, achieve synergistic killing, and reduce systemic toxicity.

### 7.2 Targeting and administration routes

Active targeting and optimized administration routes have been employed to concentrate drugs at infection sites. Mannosylation of nanostructured lipid carriers enhanced rifampicin uptake by macrophages derived from bone marrow and improved intracellular killing (Vieira et al., 2017). Stearylamine modified solid lipid nanoparticles functionalized with mannose achieved active alveolar macrophage targeting and reduced isoniazid cytotoxicity (Costa et al., 2018). A tuftsin derived peptide conjugated to rifampicin-loaded nanoparticles selectively recognized receptors on infected macrophages, doubling the anti-mycobacterial efficacy of free rifampicin (Carneiro et al., 2019). For osteoarticular tuberculosis, mannose conjugated chitosan nanoparticles loaded with rifampicin were incorporated into an injectable *in situ* gelling system, providing zero order release for over 40 h in simulated synovial fluid and enabling active macrophage targeting (Prabhu et al., 2021). PLGA nanocarriers co-encapsulating Q203, bedaquiline and superparamagnetic iron oxide nanoparticles achieved nearly 100% deposition in the pulmonary acinus under an external magnetic field and exhibited synergistic bactericidal activity against BCG (Poh et al., 2019). A codrug of ethionamide and its booster BDM43266 self-assembled into 200 nm nanoparticles. Intranasal delivery reduced the bacterial load 6-fold in a mouse model (Pastor et al., 2019). Ocular delivery of isoniazid using solid lipid nanoparticles allowed effective treatment of ocular tuberculosis (Singh et al., 2019). Pulmonary administration of cross-linked poly-β-cyclodextrin nanoparticles reduced lung bacterial burden via host-directed mechanisms (Machelart et al., 2019).

### 7.3 Intrinsic antibacterial activity, synergistic effects and challenges

Certain nanocarriers exhibit intrinsic antimicrobial activity and potentiate conventional anti-tubercular agents through synergistic mechanisms. Silver, zinc oxide and their composite nanoparticles inhibited the growth of MDR and XDR Mtb at concentrations as low as 1 μg/mL, although they were only bacteriostatic and showed cytotoxicity at high doses (Heidary et al., 2019). Green-synthesized gold-silver bimetallic nanoparticles using plant extracts exhibited potent anti-mycobacterial activity against both active and dormant bacilli (MIC <2.56 μg/mL) with high selectivity indices (94–108) and low cytotoxicity, offering a multi-target approach less prone to resistance (Singh et al., 2016). Dihydroartemisinin-loaded chitosan nanoparticles not only disrupted the cell wall but also synergized with rifampicin to overcome resistance, with an effective rate of 66.0% against clinical drug-resistant strains (Gu et al., 2021). Essential oil-based nanoemulsions possessed intrinsic antimicrobial activity and efficiently solubilized hydrophobic drugs, achieving MIC values below 0.0009 mg/mL through synergistic effects (Menon et al., 2023). Cross-linked poly-β-cyclodextrin nanoparticles exhibited intrinsic anti-mycobacterial activity by depleting plasma membrane cholesterol, disrupting lipid rafts, and inducing early apoptosis in infected macrophages, without genotoxicity (Machelart et al., 2019). Nevertheless, several challenges persist. High concentrations of silver nanoparticles exhibited significant cytotoxicity on THP-1 cells (Heidary et al., 2019), and further *in vivo* validation of safety and efficacy is required for certain nanoformulations. Future research directions should prioritize the improvement of biocompatibility, the exploration of green synthesis routes, and the integration of active targeting with synergistic drug combinations to enhance therapeutic efficacy against drug-resistant tuberculosis.

## 8 Conclusions and futuristic vision

The mycobacterial cell wall, with its unique and indispensable architecture, remains a cornerstone of anti-tuberculosis drug discovery. Over the past decades, systematic elucidation of the biosynthetic pathways of arabinogalactan, peptidoglycan, and mycolic acids has not only validated classical targets such as DprE1, InhA, and MmpL3 but also uncovered a wealth of new vulnerabilities, including DprE2, GlfT1, UGM, AftB, KasA, Pks13, FadD32, and the MurT-GatD complex. The discovery and optimization of inhibitors against these enzymes have produced a diverse array of chemical scaffolds with potent activity against drug-susceptible and multidrug-resistant *Mycobacterium tuberculosis*, several of which are advancing through clinical development. Beyond direct enzymatic inhibition, a multidimensional understanding of cell wall biology has given rise to complementary strategies, including disrupting cell wall integrity through alternative mechanisms, interfering with energy metabolism and regulatory networks, blocking the secretion and transport of essential building blocks, and harnessing host-directed therapies together with AMPs. Nanotechnology-based drug delivery systems have further empowered these efforts by overcoming the formidable permeability barrier of the mycobacterial cell wall, enabling targeted and sustained release of both existing and novel agents.

Nevertheless, key challenges persist. Resistance to new inhibitors, frequently driven by mutations in target-encoding genes, underscores the urgent need for combination regimens that simultaneously attack multiple essential pathways. Tuberculosis pathology is complex, featuring heterogeneous granulomatous lesions with variable drug penetration. Consequently, it is necessary to gain a deeper understanding of pharmacokinetics and pharmacodynamics at the infection site. Successful translation of promising preclinical candidates will require rigorous evaluation in animal models that faithfully recapitulate human disease, as well as careful optimization of dosing schedules to achieve sustained drug exposure within the lesions where bacilli reside. Detailed inhibitor information and chemical structures are available in Supplementary Table S1.

Looking forward, the future of cell wall-targeting anti-tuberculosis drug development will depend on the integration of several strategic directions. Continued discovery and validation of novel targets, particularly those involved in pathways specific to *mycobacterium* and absent in humans, will expand the therapeutic arsenal. The identification and optimization of inhibitors with improved potency, selectivity, and drug-like properties will be accelerated by the application of structure-based drug design, artificial intelligence, and machine learning. Rational design of combination therapies that target multiple essential processes holds the promise of more rapid bacterial clearance, prevention of resistance, and shortened treatment duration. Advanced drug delivery systems will be critical to overcoming the permeability barrier and achieving effective drug concentrations within granulomas. Finally, the integration of systems biology approaches will provide a holistic view of bacterial adaptation and resistance mechanisms. These approaches encompass transcriptomics, proteomics, and metabolomics, guiding the development of next-generation therapies capable of outmaneuvering pathogen evolution. Thus, exploiting the full spectrum of cell wall vulnerabilities and integrating innovative drug design and delivery technologies should enable the development of ultrashort, highly effective, and resistance-proof tuberculosis regimens. Realizing these opportunities as tangible benefits for patients worldwide will require sustained dedication to basic research, translational science, and clinical innovation.

## References

[B1] Abdelaziz R. Dube M. Mann L. Richter A. Robaa D. Reiling N. (2025). Synthesis and antimycobacterial assays of some new ethambutol analogs. Molecules 30 (3), 600. 10.3390/molecules30030600 39942704 PMC11820526

[B2] Abrahams K. A. Besra G. S. (2016). Mycobacterial cell wall biosynthesis: a multifaceted antibiotic target. Parasitology 145 (2), 116–133. 10.1017/S0031182016002377 27976597 PMC5964476

[B3] Abrahams K. A. Batt S. M. Gurcha S. S. Veerapen N. Bashiri G. Besra G. S. (2023). DprE2 is a molecular target of the anti-tubercular nitroimidazole compounds pretomanid and delamanid. Nat. Commun. 14 (1), 3828. 10.1038/s41467-023-39300-z 37380634 PMC10307805

[B4] Adewumi A. T. Elrashedy A. Soremekun O. S. Ajadi M. B. Soliman M. E. S. (2022). Weak spots inhibition in the *Mycobacterium tuberculosis* antigen 85C target for antitubercular drug design through selective irreversible covalent inhibitor-SER124. J. Biomol. Struct. Dyn. 40 (7), 2934–2954. 10.1080/07391102.2020.1844061 33155529

[B5] Ahmed D. M. Chen J. M. Sanders D. A. R. (2022). Sanders, pyrazole and triazole derivatives as *Mycobacterium tuberculosis* UDP-galactopyranose inhibitors. Pharm. (Basel) 15 (2), 197. 10.3390/ph15020197 PMC8874540 35215309

[B6] Ajin K. A. Arun Kumar S. Singh M. Akshatha H. S. Bhagyalalitha M. Pujar K. G. (2023). Novel antitubercular agents: design, synthesis, molecular dynamic and biological studies of pyrazole - 1,2,4-triazole conjugates. Chem. Biodivers. 20 (11), e202300971. 10.1002/cbdv.202300971 37882429

[B7] Alanzi A. R. Alsulais F. M. Alhaidhal B. A. (2025). A computational approach to mycolic acid biosynthesis disruption in mycobacterium tuberculosis *via* molluscan metabolites as KasA inhibitors. Sci. Rep. 15 (1), 27709. 10.1038/s41598-025-13329-0 40730846 PMC12307626

[B8] Allen R. Ames L. Baldin V. P. Butts A. Henry K. J. Durst G. (2024). An arylsulfonamide that targets cell wall biosynthesis in mycobacterium tuberculosis. *Antimicrob. Agents* . Chemother. 68 (11), e0103724. 10.1128/aac.01037-24 PMC11539219 39324799

[B9] Alsayed S. S. R. Lun S. Payne A. Bishai W. R. Gunosewoyo H. (2021). Design, synthesis and antimycobacterial evaluation of novel adamantane and adamantanol analogues effective against drug-resistant tuberculosis. Bioorg. Chem. 106, 104486. 10.1016/j.bioorg.2020.104486 33276981 PMC7775894

[B10] Altharawi A. Alossaimi M. A. Alanazi M. M. Alqahatani S. M. Tahir Ul Qamar M. (2023). An integrated computational approach towards novel drugs discovery against polyketide synthase 13 thioesterase domain of *Mycobacterium tuberculosis* . Sci. Rep. 13 (1), 7014. 10.1038/s41598-023-34222-8 37117557 PMC10147368

[B11] Ames L. Allen R. Boshoff H. I. M. Cleghorn L. A. T. Engelhart C. A. Schnappinger D. (2025). Common biological properties of *Mycobacterium tuberculosis* MmpL3 inhibitors. ACS. Infect. Dis. 11 (9), 2523–2533. 10.1021/acsinfecdis.5c00394 40827527 PMC12442057

[B12] Andrianov A. M. Furs K. V. Gonchar A. V. Skrahina A. M. Wang Y. Lyu L. D. (2025). Virtual screening and identification of promising therapeutic compounds against drug-resistant *Mycobacterium tuberculosis* β-ketoacyl-acyl carrier protein synthase I (KasA). J. Biomol. Struct. Dyn. 43 (4), 2029–2041. 10.1080/07391102.2023.2293276 38088766

[B13] Angrish N. Lalwani N. Khare G. (2023). *In silico* virtual screening for the identification of novel inhibitors against dihydrodipicolinate reductase (DapB) of *Mycobacterium tuberculosis*, a key enzyme of diaminopimelate pathway. Microbiol. Spectr. 11 (6), e0135923. 10.1128/spectrum.01359-23 37855602 PMC10714930

[B14] Armstrong T. Lamont M. Lanne A. Alderwick L. J. Thomas N. R. (2020). Inhibition of *Mycobacterium tuberculosis* InhA: design, synthesis and evaluation of new di-triclosan derivatives. Bioorg. Med. Chem. 28 (22), 115744. 10.1016/j.bmc.2020.115744 33007556

[B15] Bai L. Parkin L. A. Zhang H. Shum R. Previti M. L. Seeliger J. C. (2020). Dimethylaminophenyl hydrazides as inhibitors of the lipid transport protein LprG in mycobacteria. ACS. Infect. Dis. 6 (4), 637–648. 10.1021/acsinfecdis.9b00497 32053347 PMC7436943

[B16] Balabon O. Pitta E. Rogacki M. K. Meiler E. Casanueva R. Guijarro L. (2020). Optimization of hydantoins as potent antimycobacterial Decaprenylphosphoryl-β-d-Ribose oxidase (DprE1) inhibitors. J. Med. Chem. 63 (10), 5367–5386. 10.1021/acs.jmedchem.0c00107 32342688

[B17] Baldin V. P. Harding C. L. Quach D. Sugie J. Pogliano J. Parish T. (2025). Thienopyrimidine amide analogs target MmpL3 in mycobacterium tuberculosis. *Antimicrob. Agents* . Chemother. 69 (11), e0098025. 10.1128/aac.00980-25 PMC12587563 40980915

[B18] Berube B. Deshpande A. Bhagwat A. Parish T. (2023). Inoculum-dependent bactericidal activity of a *Mycobacterium tuberculosis* MmpL3 inhibitor. Microbiol. Read. 169 (6), 001345. 10.1099/mic.0.001345 PMC10333795 37334886

[B19] Burley K. H. Cuthbert B. J. Basu P. Newcombe J. Irimpan E. M. Quechol R. (2021). Structural and molecular dynamics of *Mycobacterium tuberculosis* malic enzyme, a potential Anti-TB drug target. ACS. Infect. Dis. 7 (1), 174–188. 10.1021/acsinfecdis.0c00735 33356117 PMC8083904

[B20] Chaitra R. Gandhi R. Jayanna N. Satyanath S. Pavadai P. Murahari M. (2023). Computational design of MmpL3 inhibitors for tuberculosis therapy. Mol. Divers. 27 (1), 357–369. 10.1007/s11030-022-10436-2 35477825

[B21] Carloni G. Gaday Q. Petit J. Martinez M. Megrian D. Sogues A. (2025). Mechanistic insights into the allosteric regulation of cell wall hydrolase RipA in *Mycobacterium tuberculosis* . bioRxiv. 10.1101/2025.06.28.662095 PMC12977500 41586520

[B22] Carneiro S. P. Carvalho K. V. de Oliveira Aguiar Soares R. D. Carneiro C. M. de Andrade M. H. G. Duarte R. S. (2019). Functionalized rifampicin-loaded nanostructured lipid carriers enhance macrophages uptake and antimycobacterial activity. Colloids. Surf. B. Biointerfaces 175, 306–313. 10.1016/j.colsurfb.2018.12.003 30553206

[B23] Chauhan A. Singh N. Kumar R. Kushwaha N. K. Prajapati V. M. Singh S. K. (2023). GlfT1 down-regulation affects *Mycobacterium tuberculosis* biofilm formation and its *in-vitro* and *in-vivo* survival. Tuberc. (Edinb) 141, 102352. 10.1016/j.tube.2023.102352 37267752

[B24] Chengalroyen M. D. Jordaan A. Seldon R. Ioerger T. Franzblau S. G. Nasr M. (2020). Biological profiling enables rapid mechanistic classification of phenotypic screening hits and identification of KatG activation-dependent pyridine carboxamide prodrugs with activity against *Mycobacterium tuberculosis* . Front. Cell. Infect. Microbiol. 10, 582416. 10.3389/fcimb.2020.582416 33282750 PMC7691319

[B25] Chhabra S. Kumar S. Parkesh R. (2021). Chemical space exploration of DprE1 inhibitors using chemoinformatics and artificial intelligence. ACS. Omega. 6 (22), 14430–14441. 10.1021/acsomega.1c01314 34124465 PMC8190903

[B26] Choksi H. Carbone J. Paradis N. J. Bennett L. Bui-Linh C. Wu C. (2024). Novel inhibitors to MmpL3 transporter of *Mycobacterium tuberculosis* by structure-based high-throughput virtual screening and molecular dynamics simulations. ACS. Omega. 9 (12), 13782–13796. 10.1021/acsomega.3c08401 38559933 PMC10976370

[B27] Chu J. Koirala B. Forelli N. Vila-Farres X. Ternei M. A. Ali T. (2020). Synthetic-bioinformatic natural product antibiotics with diverse modes of action. J. Am. Chem. Soc. 142 (33), 14158–14168. 10.1021/jacs.0c04376 32697091 PMC8011376

[B28] Costa A. Sarmento B. Seabra V. (2018). Mannose-functionalized solid lipid nanoparticles are effective in targeting alveolar macrophages. Eur. J. Pharm. Sci. 114, 103–113. 10.1016/j.ejps.2017.12.006 29229273

[B29] Cui Y. Lanne A. Peng X. Browne E. Bhatt A. Coltman N. J. (2024). Azetidines kill multidrug-resistant *Mycobacterium tuberculosis* without detectable resistance by blocking mycolate assembly. J. Med. Chem. 67 (4), 2529–2548. 10.1021/acs.jmedchem.3c01643 38331432 PMC10895678

[B30] Cui Y. Lanne A. Avula S. Hama Salih M. A. Peng X. Milne G. (2025). Discovery of novel fluorescent amino-pyrazolines that detect and kill mycobacterium. Tuberculosis. Eur. J. Med. Chem. 297, 117889. 10.1016/j.ejmech.2025.117889 40592183

[B31] Daffé M. Marrakchi H. (2019). Unraveling the structure of the mycobacterial envelope. Microbiol. Spectr. 7 (4). 10.1128/microbiolspec.GPP3-0027-2018 31267927 PMC10957186

[B32] Das N. Jena P. K. Pradhan S. K. (2020). Arabinosyltransferase C enzyme of *Mycobacterium tuberculosis*, a potential drug target: an insight from molecular docking study. Heliyon 6 (2), e02693. 10.1016/j.heliyon.2019.e02693 32090179 PMC7026281

[B33] de Castro R. R. do Carmo F. A. Martins C. Simon A. de Sousa V. P. Rodrigues C. R. (2021). Clofazimine functionalized polymeric nanoparticles for brain delivery in the tuberculosis treatment. Int. J. Pharm. 602, 120655. 10.1016/j.ijpharm.2021.120655 33915184

[B34] de Munnik M. Calvopiña K. Rabe P. Schofield C. J. (2025). Targeting mycobacterial transpeptidases: evaluating the roles of Ldt and PBP inhibition in suppressing Mycobacterium smegmatis. Antimicrob. Agents. Chemother. 69 (10), e0012625. 10.1128/aac.00126-25 40952390 PMC12486854

[B35] Diab A. Dickerson H. Al Musaimi O. (2025). Targeting the heart of mycobacterium: advances in anti-tubercular agents disrupting cell wall biosynthesis. Pharmaceuticals 18 (1), 70. 10.3390/ph18010070 39861133 PMC11768153

[B36] Dos Santos Silva C. Dias M. V. B. (2025). The multiple roles of the NlpC_P60 peptidase family in mycobacteria – an underexplored target for antimicrobial drug discovery. FEBS. Lett. 599 (9), 1203–1221. 10.1002/1873-3468.70021 40028658 PMC12067865

[B37] Dousa K. M. Nguyen D. C. Kurz S. G. Taracila M. A. Bethel C. R. Schinabeck W. (2022). Inhibiting Mycobacterium abscessus cell wall synthesis: using a novel diazabicyclooctane β-Lactamase inhibitor to augment β-Lactam action. mBio 13 (1), e0352921. 10.1128/mbio.03529-21 35073757 PMC8787486

[B38] Dube P. S. Legoabe L. J. Jordaan A. Sigauke L. Warner D. F. Beteck R. M. (2023). Quinolone analogues of benzothiazinone: synthesis, antitubercular structure-activity relationship and ADME profiling. Eur. J. Med. Chem. 258, 115539. 10.1016/j.ejmech.2023.115539 37321107

[B39] Dulberger C. L. Rubin E. J. Boutte C. C. (2019). The mycobacterial cell envelope — a moving target. Nat. Rev. Microbiol. 18 (1), 47–59. 10.1038/s41579-019-0273-7 31728063

[B40] Eckhardt E. Li Y. Mamerow S. Schinköthe J. Sehl-Ewert J. Dreisbach J. (2023). Pharmacokinetics and efficacy of the benzothiazinone BTZ-043 against tuberculous mycobacteria inside granulomas in the Guinea pig model. Antimicrob. Agents. Chemother. 67 (4), e0143822. 10.1128/aac.01438-22 36975792 PMC10112198

[B41] El Haddoumi G. Mansouri M. Kourou J. Belyamani L. Ibrahimi A. Kandoussi I. (2024). Targeting decaprenylphosphoryl-β-D-ribose 2′-epimerase for innovative drug development against Mycobacterium tuberculosis drug-resistant strains. Bioinform. Biol. Insights 18, 11779322241257039. 10.1177/11779322241257039 38812740 PMC11135120

[B42] El-Ridy M. S. Yehia S. A. Kassem M. A. Mostafa D. M. Nasr E. A. Asfour M. H. (2015). Niosomal encapsulation of ethambutol hydrochloride for increasing its efficacy and safety. Drug. Deliv. 22 (1), 21–36. 10.3109/10717544.2013.868556 24359403

[B43] Eldehna W. M. El Hassab M. A. Abdelshafi N. A. Al-Zahraa Sayed F. Fares M. Al-Rashood S. T. (2022). Development of potent nanosized isatin-isonicotinohydrazide hybrid for management of *Mycobacterium tuberculosis* . Int. J. Pharm. 612, 121369. 10.1016/j.ijpharm.2021.121369 34906651

[B44] Erkin A. V. Serebryakov E. B. Krutikov V. I. (2023). 2-[(2-Amino-6-methylpyrimidin-4-yl)sulfanyl]-N-arylacetamides: discovery of a new class of anti-tubercular agents and prospects for their further structural modification. Bioorg. Med. Chem. Lett. 83, 129189. 10.1016/j.bmcl.2023.129189 36805047

[B45] Faïon L. Djaout K. Frita R. Pintiala C. Cantrelle F. X. Moune M. (2020). Discovery of the first *Mycobacterium tuberculosis* MabA (FabG1) inhibitors through a fragment-based screening. Eur. J. Med. Chem. 200, 112440. 10.1016/j.ejmech.2020.112440 32505086

[B46] Fan D. Wang B. Stelitano G. Savková K. Shi R. Huszár S. (2021). Structural and activity relationships of 6-Sulfonyl-8-Nitrobenzothiazinones as antitubercular agents. J. Med. Chem. 64 (19), 14526–14539. 10.1021/acs.jmedchem.1c01049 34609861

[B47] Fan D. Wang B. Stelitano G. Savková K. Riabova O. Shi R. (2023). Side chain-modified benzothiazinone derivatives with anti-mycobacterial activity. Biomedicines 11 (7), 1975. 10.3390/biomedicines11071975 37509615 PMC10377601

[B48] Fang C. Zhang H. He J. Tian X. Zeng S. Han X. (2025). “GrcC1 mediates low-level resistance to multiple drugs,”. Editors marinum M. abscessus M. , 13. 10.1128/spectrum.02289-24 PMC11960048 40009796

[B49] Finger V. Kucera T. Kafkova R. Muckova L. Dolezal R. Kubes J. (2023). 2,6-Disubstituted 7-(naphthalen-2-ylmethyl)-7H-purines as a new class of potent antitubercular agents inhibiting DprE1. Eur. J. Med. Chem. 258, 115611. 10.1016/j.ejmech.2023.115611 37421887

[B50] Flint L. Korkegian A. Parish T. (2020). InhA inhibitors have activity against non-replicating *Mycobacterium tuberculosis* . PLoS. One. 15 (11), e0239354. 10.1371/journal.pone.0239354 33201882 PMC7671525

[B51] Ge Y. Luo Q. Liu L. Shi Q. Zhang Z. Yue X. (2024). S288T mutation altering MmpL3 periplasmic domain channel and H-bond network: a novel dual drug resistance mechanism. J. Mol. Model. 30 (2), 39. 10.1007/s00894-023-05814-y 38224406

[B52] Green S. R. Harrison J. R. Thompson S. Murugesan D. Libardo M. D. J. Engelhart C. A. (2025). Identification of a series containing a pentafluorophenyl moiety that targets Pks13 to inhibit growth of *Mycobacterium tuberculosis* . ACS. Infect. Dis. 11 (3), 715–726. 10.1021/acsinfecdis.4c00808 40014668 PMC11915372

[B53] Gu X. Cheng Q. He P. Zhang Y. Jiang Z. Zeng Y. (2021). Dihydroartemisinin-loaded chitosan nanoparticles inhibit the rifampicin-resistant *Mycobacterium tuberculosis* by disrupting the cell wall. Front. Microbiol. 12, 735166. 10.3389/fmicb.2021.735166 34630358 PMC8500176

[B54] Guy C. S. Cooper C. Karlikowska M. Harrison J. Singh A. Servín-González L. S. (2025). The deacetylase NagA mediates the remodeling and recycling of peptidoglycan-derived amino sugars in mycobacteria. J. Biol. Chem. 301 (11), 110597. 10.1016/j.jbc.2025.110597 40818611 PMC12617635

[B55] Han X. Chen C. Wang H. Kang J. Yan Q. Ma Y. (2022). GlmU inhibitor from the roots of Euphorbia ebracteolata as an anti-tuberculosis agent. RSC. Adv. 12 (28), 18266–18273. 10.1039/d2ra02044k 35800323 PMC9214920

[B56] Hariguchi N. Chen X. Hayashi Y. Kawano Y. Fujiwara M. Matsuba M. (2020). OPC-167832, a novel carbostyril derivative with potent antituberculosis activity as a DprE1 inhibitor. Antimicrob. Agents. Chemother. 64 (6), e02020. 10.1128/AAC.02020-19 32229496 PMC7269503

[B57] Healy C. Gouzy A. Ehrt S. (2020). Peptidoglycan hydrolases RipA and Ami1 are critical for replication and persistence of *Mycobacterium tuberculosis* in the host. mBio 11 (2), e03315–e03319. 10.1128/mBio.03315-19 32127458 PMC7064781

[B58] Heidary M. Zaker Bostanabad S. Amini S. M. Jafari A. Ghalami Nobar M. Ghodousi A. (2019). The anti-mycobacterial activity of Ag, ZnO, and Ag- ZnO nanoparticles against MDR- and XDR-mycobacterium tuberculosis. Infect. Drug. Resist. 12, 3425–3435. 10.2147/IDR.S221408 31807033 PMC6839584

[B59] Hu T. Yang X. Liu F. Sun S. Xiong Z. Liang J. (2022). Structure-based design of anti-mycobacterial drug leads that target the mycolic acid transporter MmpL3. Structure 30 (10), 1395–1402.e4. 10.1016/j.str.2022.07.009 35981536

[B60] Huang F. Shi M. Chen L. Li X. Wang Z. Jiang D. (2025). ACA kills *Mycobacterium tuberculosis* and M. bovis by targeting cell wall core assembling protein CpsA. Commun. Biol. 8 (1), 1706. 10.1038/s42003-025-09107-3 41299017 PMC12658130

[B61] Hwang S. Heo B. E. Nguyen T. Q. Kim Y. J. Lee S. G. Huynh T. H. (2025). Arenicolide family macrolides provide a new therapeutic lead combating multidrug-resistant tuberculosis. Angew. Chem. Int. Ed. Engl. 64 (1), e202412994. 10.1002/anie.202412994 39400949 PMC11701349

[B62] Ibrahim M. A. A. Mahmoud D. G. M. Abdelrahman A. H. M. Abdeljawaad K. A. A. Mekhemer G. A. H. Shoeib T. (2024). Benzothiazinone analogs as anti-mycobacterium tuberculosis DprE1 irreversible inhibitors: covalent docking, validation, and molecular dynamics simulations. Plos. One. 19 (11), e0314422. 10.1371/journal.pone.0314422 39585898 PMC11588222

[B63] Jiang S. Li H. Zhang L. Mu W. Zhang Y. Chen T. (2025). Generic diagramming platform (GDP): a comprehensive database of high-quality biomedical graphics. Nucleic. Acids. Res. 53 (D1), D1670–D1676. 10.1093/nar/gkae973 39470721 PMC11701665

[B64] Johnson W. C. Alivisatos A. Smith T. C. 2nd Van N. Soni V. Wallach J. B. (2025). Integration of multi-modal measurements identifies critical mechanisms of tuberculosis drug action. Cell 16 (8), 101348. 10.1016/j.cels.2025.101348 PMC12365861 40738114

[B65] Kamera S. Sharma V. K. Prasad V. B. Garlapati A. (2024). Identification of potential inhibitors of Mtb InhA: a pharmacoinformatics approach. J. Biomol. Struct. Dyn. 42 (15), 7957–7971. 10.1080/07391102.2023.2242499 37526169

[B66] Kapp E. Joubert J. Sampson S. L. Warner D. F. Seldon R. Jordaan A. (2021). Antimycobacterial activity, synergism, and mechanism of action evaluation of novel polycyclic amines against *Mycobacterium tuberculosis* . Adv. Pharmacol. Pharm. Sci. 2021, 5583342–5583348. 10.1155/2021/5583342 34240057 PMC8238621

[B67] Kaur G. Mehta S. K. Kumar S. Bhanjana G. Dilbaghi N. (2015). Coencapsulation of hydrophobic and hydrophilic antituberculosis drugs in synergistic Brij 96 microemulsions: a biophysical characterization. J. Pharm. Sci. 104 (7), 2203–2212. 10.1002/jps.24469 25951802

[B68] Kb S. Kumari A. Shetty D. Fernandes E. Dv C. Jays J. (2020). Structure based pharmacophore modelling approach for the design of azaindole derivatives as DprE1 inhibitors for tuberculosis. J. Mol. Graph. Model. 101, 107718. 10.1016/j.jmgm.2020.107718 32949960

[B69] Khatak S. Mehta M. Awasthi R. Paudel K. R. Singh S. K. Gulati M. (2020). Solid lipid nanoparticles containing anti-tubercular drugs attenuate the Mycobacterium marinum infection. Tuberc. (Edinb) 125, 102008. 10.1016/j.tube.2020.102008 33059322

[B70] Khonde L. P. Müller R. Boyle G. A. Reddy V. Nchinda A. T. Eyermann C. J. (2021). 1,3-Diarylpyrazolyl-acylsulfonamides as potent anti-tuberculosis agents targeting cell wall biosynthesis in *Mycobacterium tuberculosis* . J. Med. Chem. 64 (17), 12790–12807. 10.1021/acs.jmedchem.1c00837 34414766 PMC10500703

[B71] Klevesath L. Noschka R. Vomhof T. Mohnani J. Grieshober M. Michaelis J. (2025). RapTB: a lung-derived hemoglobin fragment with activity against *Mycobacterium tuberculosis* . Front. Microbiol. 16, 1669022. 10.3389/fmicb.2025.1669022 41209728 PMC12592065

[B72] Knoll K. E. Lindeque Z. Adeniji A. A. Oosthuizen C. B. Lall N. Loots D. T. (2021). Elucidating the antimycobacterial mechanism of action of decoquinate derivative RMB041 using metabolomics. Antibiot. (Basel) 10 (6), 693. 10.3390/antibiotics10060693 PMC8228794 34200519

[B73] Kotliarova M. S. Shumkov M. S. Goncharenko A. V. (2024). Toward *Mycobacterium tuberculosis* virulence inhibition: beyond cell wall. Microorganisms 13 (1), 21. 10.3390/microorganisms13010021 39858789 PMC11767696

[B74] Krieger I. V. Sukheja P. Yang B. Tang S. Selle D. Woods A. (2025). SuFEx-based antitubercular compound irreversibly inhibits Pks13. Nature 645 (8081), 755–763. 10.1038/s41586-025-09286-3 40739353 PMC12962449

[B75] Kuldeep J. Sharma S. K. Sharma T. Singh B. N. andSiddiqi M. I. (2021). Targeting Mycobacterium tuberculosis enoyl-acyl carrier protein reductase using computational tools for identification of potential inhibitor and their biological activity. Mol. Inf. 40 (5), e2000211. 10.1002/minf.202000211 33283460

[B76] Kumar P. Saumya K. U. Giri R. (2020). Identification of peptidomimetic compounds as potential inhibitors against MurA enzyme of *Mycobacterium tuberculosis* . J. Biomol. Struct. Dyn. 38 (17), 4997–5013. 10.1080/07391102.2019.1696231 31755364

[B77] Kumar N. Srivastava R. Mongre R. K. Mishra C. B. Kumar A. Khatoon R. (2022). Identifying the novel inhibitors against the mycolic acid biosynthesis pathway target “mtFabH” of *Mycobacterium tuberculosis* . Front. Microbiol. 13, 818714. 10.3389/fmicb.2022.818714 35602011 PMC9121832

[B78] Kumbalathara A. D. U. S. S. Bartolomeu Halicki P. C. Kalera K. Swarts B. M. Rohde K. H. Sucheck S. J. (2025). Synthesis and evaluation of Trehalose-Pks13 inhibitor conjugates targeting mycobacteria. Carbohydr. Res. Carbohydr. Res. 553, 109506. 10.1016/j.carres.2025.109506 40359660 PMC12406997

[B79] Le N. H. Constant P. Tranier S. Nahoum V. Guillet V. Maveyraud L. (2022). Drug screening approach against mycobacterial fatty acyl-AMP ligase FAAL32 renews the interest of the salicylanilide pharmacophore in the fight against tuberculosis. Bioorg. Med. Chem. 71, 116938. 10.1016/j.bmc.2022.116938 35933838

[B80] Li W. Yan Z. Zhang N. Zhang Z. Xiang X. (2023). Novel role of PE_PGRS47 in the alteration of mycobacterial cell wall integrity and drug resistance. Arch. Microbiol. 205 (5), 174. 10.1007/s00203-023-03515-x 37022460

[B81] Liang J. Liu Y. Guan Q. Li Y. Zheng M. Z. Zhang X. L. (2025). Discovery of novel pyrimidinetrione derivatives as DprE1 inhibitors with potent antimycobacterial activities. Eur. J. Med. Chem. 289, 117416. 10.1016/j.ejmech.2025.117416 39999693

[B82] Linhares L. A. Dos Santos Peixoto A. Correia de Sousa L. A. Lucena Laet J. P. da Silva Santos A. C. Alves Pereira V. R. (2023). *In vitro* bioevaluation and docking study of dihydrosphingosine and ethambutol analogues against sensitive and multi-drug resistant *Mycobacterium tuberculosis* . Eur. J. Med. Chem. 258, 115579. 10.1016/j.ejmech.2023.115579 37399709

[B83] Liu Y. Brown C. M. Erramilli S. Su Y. C. Guu S. Y. Tseng P. S. (2025). Structural insights into terminal arabinosylation of mycobacterial cell wall arabinan. Nat. Commun. 16 (1), 3973. 10.1038/s41467-025-58196-5 40301320 PMC12041299

[B84] Liu T. Meng J. Wang B. Li X. Wang Q. Liu S. (2025). Identification of BMVC-8C3O as a novel Pks13 inhibitor with anti-tuberculosis activity. Tuberc. (Edinb) 150, 102579. 10.1016/j.tube.2024.102579 39579511

[B85] Lun S. Xiao S. Zhang W. Wang S. Gunosewoyo H. Yu L. F. (2023). Therapeutic potential of coumestan Pks13 inhibitors for tuberculosis. Antimicrob. Agents. Chemother. 95 (5), e02190. 10.1128/AAC.02190-20 33558290 PMC8092898

[B86] Ma Y. Nieto-Fabregat F. Fan H. Xian Q. Takahashi M. Olmeo F. (2025). Chemical synthesis of Arabinogalactans from the *Mycobacterium tuberculosis* cell wall up to the 92-mer and structure-conformation-activity relationship studies. Angew. Chem. Int. Ed. Engl. 64 (48), e202515896. 10.1002/anie.202515896 41090462

[B87] Maaliki C. Fu J. Villaume S. Viljoen A. Raynaud C. Hammoud S. (2020). Synthesis and evaluation of heterocycle structures as potential inhibitors of *Mycobacterium tuberculosis* UGM. Bioorg. Med. Chem. 28 (13), 115579. 10.1016/j.bmc.2020.115579 32546296

[B88] Machelart A. Salzano G. Li X. Demars A. Debrie A. S. Menendez-Miranda M. (2019). Intrinsic antibacterial activity of nanoparticles made of beta-Cyclodextrins potentiates their effect as drug nanocarriers against tuberculosis. ACS. Nano. 13 (4), 3992–4007. 10.1021/acsnano.8b07902 30822386 PMC6718168

[B89] Madacki J. Kopál M. Jackson M. Korduláková J. (2021). Mycobacterial epoxide hydrolase EphD is inhibited by urea and thiourea derivatives. Int. J. Mol. Sci. 22 (6), 2884. 10.3390/ijms22062884 33809178 PMC7998700

[B90] Madani A. Mallick I. Guy A. Crauste C. Durand T. Fourquet P. (2020). Dissecting the antibacterial activity of oxadiazolone-core derivatives against Mycobacterium abscessus. PLoS. One. 15 (9), e0238178. 10.1371/journal.pone.0238178 32946441 PMC7500638

[B91] Maddipatla S. Agnivesh P. K. Bakchi B. Nanduri S. Kalia N. P. Yaddanapudi V. M. (2025). New pyrazole-based derivatives targeting MmpL3 transporter in mycobacterium tuberculosis: design, synthesis, biological evaluation and molecular docking studies. Mol. Divers. 29 (6), 6437–6458. 10.1007/s11030-025-11152-3 40085403

[B92] Maitra A. Nukala S. Dickman R. Martin L. T. Munshi T. Gupta A. (2021). Characterization of the MurT/GatD complex in *Mycobacterium tuberculosis* towards validating a novel anti-tubercular drug target. JAC. Antimicrob. Resist. 3 (1), dlab028. 10.1093/jacamr/dlab028 34223102 PMC8210147

[B93] Mali S. N. Pandey A. Bhandare R. R. Shaik A. B. (2022). Identification of hydantoin based Decaprenylphosphoryl-β-d-Ribose oxidase (DprE1) inhibitors as antimycobacterial agents using computational tools. Sci. Rep. 12 (1), 16368. 10.1038/s41598-022-20325-1 36180452 PMC9525719

[B94] Malík I. Čižmárik J. Kováč G. Pecháčová M. Hudecova L. (2021). Telacebec (Q203): is there a novel effective and safe anti-tuberculosis drug on the horizon? Ceska. Slov. Farm. 70 (5), 164–171. 10.5817/CSF2021-5-164 35114793

[B95] Malwal S. R. Mazurek B. Ko J. Xie P. Barnes C. Varvitsiotis C. (2023). Investigation into the mechanism of action of the tuberculosis drug candidate SQ109 and its metabolites and analogues in mycobacteria. J. Med. Chem. 66 (11), 7553–7569. 10.1021/acs.jmedchem.3c00398 37235809 PMC10330530

[B96] Manthattil Vysyan S. Suraj Prasanna M. Jayanandan A. Gangadharan A. K. Chittalakkottu S. (2024). Phytocompounds hesperidin, rebaudioside a and rutin as drug leads for the treatment of tuberculosis targeting mycobacterial phosphoribosyl pyrophosphate synthetase. J. Biomol. Struct. Dyn. 44 (1), 316–330. 10.1080/07391102.2024.2438363 39659199

[B97] McNeil M. B. O'Malley T. Dennison D. Shelton C. D. Sunde B. Parish T. (2020). Multiple mutations in *Mycobacterium tuberculosis* MmpL3 increase resistance to MmpL3 inhibitors. mSphere 5 (5), e00985. 10.1128/mSphere.00985-20 33055263 PMC7565900

[B98] Menon P. M. Chandrasekaran N. C. G. P. D. Shanmugam S. (2023). Multi-drug loaded eugenol-based nanoemulsions for enhanced anti-mycobacterial activity. RSC. Med. Chem. 14 (3), 433–443. 10.1039/d2md00320a 36970149 PMC10034140

[B99] Modi P. Patel S. Chhabria M. (2023). Discovery of newer pyrazole derivatives with potential anti-tubercular activity *via* 3D-QSAR based pharmacophore modelling, virtual screening, molecular docking and molecular dynamics simulation studies. Mol. Divers. 27 (4), 1547–1566. 10.1007/s11030-022-10511-8 35969333

[B100] Mostert D. Braun J. Zimmerman M. D. Engelhart C. A. Berndl S. Quoika P. K. (2025). Tailored phenyl ureas eradicate drug-resistant *Mycobacterium tuberculosis* by targeting mycolic acid cell wall assembly. Chem. Sci. 16 (21), 9472–9483. 10.1039/d5sc02565f 40313523 PMC12041881

[B101] Mubarak M. M. Kantroo H. A. Mir F. A. Kumar C. Ahmad Z. (2025). Targeting InhA in drug-resistant mycobacterium tuberculosis: potent antimycobacterial activity of diaryl ether dehydrozingerone derivatives. Arch. Microbiol. 207 (2), 34. 10.1007/s00203-025-04238-x 39812792

[B102] Naidoo T. J. Senzani S. Singh R. Pillay B. Pillay M. (2025). *Mycobacterium tuberculosis* curli pili (MTP) and heparin-binding hemagglutinin adhesin (HBHA) facilitate regulation of central carbon metabolism, enhancement of ATP synthesis and cell wall biosynthesis. Arch. Microbiol. 207 (7), 156. 10.1007/s00203-025-04352-w 40437078 PMC12119724

[B103] Nantongo M. Nguyen D. C. Bethel C. R. Taracila M. A. Li Q. Dousa K. M. (2024). Durlobactam, a diazabicyclooctane β-Lactamase inhibitor, inhibits BlaC and peptidoglycan transpeptidases of *Mycobacterium tuberculosis* . ACS. Infect. Dis. 10 (5), 1767–1779. 10.1021/acsinfecdis.4c00119 38619138

[B104] Nantongo M. Nguyen D. C. Shin E. Bethel C. R. Taracila M. A. Dousa K. M. (2025). Exploring β-lactam interactions with DacB1: unraveling optimal therapies for combating drug-resistant *Mycobacterium tuberculosis* . mBio. 16 (8), e0137225. 10.1128/mbio.01372-25 40637416 PMC12345272

[B105] Nemati E. Mokhtarzadeh A. Panahi-Azar V. Mohammadi A. Hamishehkar H. Mesgari-Abbasi M. (2019). Ethambutol-loaded solid lipid nanoparticles as dry powder inhalable formulation for tuberculosis therapy. AAPS. PharmSciTech. 20 (3), 120. 10.1208/s12249-019-1334-y 30796625

[B106] Niranjan K. Srivastava R. Prakash A. Lynn A. M. (2021). Virtual screening and free energy estimation for identifying *Mycobacterium tuberculosis* flavoenzyme DprE1 inhibitors. J. Mol. Graph. Model. 102, 107770. 10.1016/j.jmgm.2020.107770 33065513

[B107] Obadawo B. S. Bartolomeu Halicki P. C. Becker K. L. Seeliger J. C. Rohde K. H. Sucheck S. J. (2025). Discovery of 2,4,5-Substituted benzoxazole derivatives as Pks13 inhibitors *via* the scaffold hopping strategy. ACS. Infect. Dis. 11 (6), 1460–1472. 10.1021/acsinfecdis.4c01054 40338207 PMC12977085

[B108] Onajole O. K. Lun S. Yun Y. J. Langue D. Y. Jaskula-Dybka M. Flores A. (2020). Design, synthesis, and biological evaluation of novel imidazo[1,2-a]pyridinecarboxamides as potent anti-tuberculosis agents. Chem. Biol. Drug. Des. 96 (6), 1362–1371. 10.1111/cbdd.13739 32515129 PMC8720286

[B110] Panda D. Maharana J. Sharma A. Wadavrao S. B. Chowdhury A. Laskar M. A. (2026). Identifying potent inhibitors for *Mycobacterium tuberculosis* MabA (FabG1). Mol. Divers. 30 (1), 837–850. 10.1007/s11030-025-11205-7 40358829

[B111] Pastor A. Machelart A. Li X. Willand N. Baulard A. Brodin P. (2019). A novel codrug made of the combination of ethionamide and its potentiating booster: synthesis, self-assembly into nanoparticles and antimycobacterial evaluation. Org. Biomol. Chem. 17 (20), 5129–5137. 10.1039/c9ob00680j 31073555

[B112] Pawar A. Jha P. Chopra M. Chaudhry U. Saluja D. (2020). Screening of natural compounds that targets glutamate racemase of *Mycobacterium tuberculosis* reveals the anti-tubercular potential of flavonoids. Sci. Rep. 10 (1), 949. 10.1038/s41598-020-57658-8 31969615 PMC6976638

[B113] Pflégr V. Stolaříková J. Pál A. Korduláková J. Krátký M. (2023). Novel pyrimidine-1,3,4-oxadiazole hybrids and their precursors as potential antimycobacterial agents. Future. Med. Chem. 15 (12), 1049–1067. 10.4155/fmc-2023-0096 37555280

[B114] Pflégr V. Horváth L. Stolaříková J. Pál A. Korduláková J. Bősze S. (2021). Design and synthesis of 2-(2-isonicotinoylhydrazineylidene)propanamides as InhA inhibitors with high antitubercular activity. Eur. J. Med. Chem. 223, 113668. 10.1016/j.ejmech.2021.113668 34198149

[B115] Pflégr V. Stolaříková J. Karabanovich G. Maixnerová J. Pál A. Korduláková J. (2025). 5-(3,5-Dinitrophenyl)-1,3,4-oxadiazol-2-amine derivatives, their precursors, and analogues: synthesis and evaluation of novel highly potent antitubercular agent. PLOS. One. 20 (5), e0324608. 10.1371/journal.pone.0324608 40440331 PMC12121777

[B116] Poh W. Ab Rahman N. Ostrovski Y. Sznitman J. Pethe K. Loo S. C. J. (2019). Active pulmonary targeting against tuberculosis (TB) *via* triple-encapsulation of Q203, bedaquiline and superparamagnetic iron oxides (SPIOs) in nanoparticle aggregates. Drug. Deliv. 26 (1), 1039–1048. 10.1080/10717544.2019.1676841 31691600 PMC6844420

[B117] Polinário G. Rosa M. A. B. C. Campos D. L. Moraes L. L. S. de Campos M. M. A. Silva I. G. M. (2025). Tanshinones target drug-resistant tuberculosis: efficacy, selectivity, and potential mechanism of action. RSC. Med. Chem. 16 (12), 6020–6030. 10.1039/d5md00637f 41063792 PMC12503125

[B118] Pornprom T. Pakamwong B. Sukchit D. Yuguchi M. Nagura Y. Sabishiro H. (2025). Designing novel InhA inhibitors for antituberculosis agents using *ab initio* fragment molecular orbital calculations. ACS. Omega. 10 (27), 29547–29557. 10.1021/acsomega.5c02912 40687024 PMC12268426

[B119] Prabhu P. Fernandes T. Chaubey P. Kaur P. Narayanan S. Vk R. (2021). Mannose-conjugated chitosan nanoparticles for delivery of rifampicin to osteoarticular tuberculosis. Drug. Deliv. Transl. Res. 11 (4), 1509–1519. 10.1007/s13346-021-01003-7 34021478

[B120] Ramey M. E. Kaya F. Bauman A. A. Massoudi L. M. Sarathy J. P. Zimmerman M. D. (2023). Drug distribution and efficacy of the DprE1 inhibitor BTZ-043 in the C3HeB/FeJ mouse tuberculosis model. Antimicrob. Agents. Chemother. 67 (11), e0059723. 10.1128/aac.00597-23 37791784 PMC10648937

[B121] Rani N. Rajmani R. S. Surolia A. (2025). Identification of an isoxazole derivative as an antitubercular compound for targeting the FadD enzymes of *Mycobacterium tuberculosis* . J. Med. Chem. 68 (1), 270–286. 10.1021/acs.jmedchem.4c01844 39693602

[B122] Ravichandran R. Ridzwan N. F. W. Mohamad S. B. (2022). Ensemble-based high-throughput virtual screening of natural ligands using the super Natural-II database against cell-wall protein dTDP-4-dehydrorhamnose reductase (RmlD) in *Mycobacterium tuberculosis* . J. Biomol. Struct. Dyn. 40 (11), 5069–5078. 10.1080/07391102.2020.1867641 33382017

[B123] Robertson G. T. Ramey M. E. Massoudi L. M. Carter C. L. Zimmerman M. Kaya F. (2021). Comparative analysis of pharmacodynamics in the C3HeB/FeJ mouse tuberculosis model for DprE1 inhibitors TBA-7371, PBTZ169, and OPC-167832. Antimicrob. Agents. Chemother. 65 (11), e0058321. 10.1128/AAC.00583-21 34370580 PMC8522729

[B124] Saifullah B. Maitra A. Chrzastek A. Naeemullah B. Fakurazi S. Bhakta S. (2017). Nano-formulation of ethambutol with multifunctional graphene oxide and magnetic nanoparticles retains its anti-tubercular activity with prospects of improving chemotherapeutic efficacy. Molecules 22 (10), 1697. 10.3390/molecules22101697 29023384 PMC6151652

[B125] Salgueiro-Toledo V. C. Bertol J. Gutierrez C. Serrano-Mestre J. L. Ferrer-Luzon N. Vázquez-Iniesta L. (2025). Maintenance of cell wall remodeling and vesicle production are connected in *Mycobacterium tuberculosis* . Elife 13, RP94982. 10.7554/eLife.94982 39960848 PMC11832169

[B126] Samoon R. Sau S. Roy A. Parida K. K. Sharma K. Yakkala P. A. (2024). Development and evaluation of bis-benzothiazoles as a new class of benzothiazoles targeting DprE1 as antitubercular agents. ACS. Infect. Dis. 10 (9), 3320–3331. 10.1021/acsinfecdis.4c00415 39150887

[B127] Shaku M. T. Ocius K. L. Apostolos A. J. Pires M. M. VanNieuwenhze M. S. Dhar N. (2023). Amidation of glutamate residues in mycobacterial peptidoglycan is essential for cell wall cross-linking. Front. Cell. Infect. Microbiol. 13, 1205829. 10.3389/fcimb.2023.1205829 37692163 PMC10484409

[B128] Shi R. Wang B. Stelitano G. Wu X. Shan Y. Wu Y. (2022). Development of 6-Methanesulfonyl-8-nitrobenzothiazinone based antitubercular agents. ACS. Med. Chem. Lett. 13 (4), 593–598. 10.1021/acsmedchemlett.1c00652 35450361 PMC9014434

[B129] Singh R. Nawale L. Arkile M. Wadhwani S. Shedbalkar U. Chopade S. (2016). Phytogenic silver, gold, and bimetallic nanoparticles as novel antitubercular agents. Int. J. Nanomedicine. 11, 1889–1897. 10.2147/IJN.S102488 27217751 PMC4862349

[B130] Singh M. Guzman-Aranguez A. Hussain A. Srinivas C. S. Kaur I. P. (2019). Solid lipid nanoparticles for ocular delivery of isoniazid: evaluation, proof of concept and *in vivo* safety and kinetics. Nanomedicine (Lond). 14 (4), 465–491. 10.2217/nnm-2018-0278 30694726

[B131] Singh M. Batt S. M. Canales C. S. C. Pavan F. R. Kumar S. A. Akshatha H. S. (2024). Novel hybrids of 1,2,3-triazole-benzoxazole: design, synthesis, and assessment of DprE1 enzyme inhibitors using fluorometric assay and computational analysis. J. Enzyme. Inhib. Med. Chem. 39 (1), 2403744. 10.1080/14756366.2024.2403744 39329328 PMC11441021

[B132] Snehalatha A. V. Kumar N. V. A. (2025). A computational approach to repurposing natural products for DprE1 inhibition. Sci. (Cairo) 2025, 2105236. 10.1155/sci5/2105236 PMC12267967 40678077

[B133] Sodani M. Misra C. S. Nigam G. Fatima Z. Kulkarni S. Rath D. (2024). MSMEG_0311 is a conserved essential polar protein involved in mycobacterium cell wall metabolism. Int. J. Biol. Macromol. 260 (Pt 2), 129583. 10.1016/j.ijbiomac.2024.129583 38242409

[B134] Sundar S. Thangamani L. Piramanayagam S. Natarajan J. (2021). Discovering mycobacterial lectins as potential drug targets and vaccine candidates for tuberculosis treatment: a theoretical approach. J. Proteins. Proteom. 12 (2), 93–104. 10.1007/s42485-021-00065-y 34025063 PMC8129965

[B135] Sundararajan S. Karunakaran K. Muniyan R. (2024). Structure based virtual screening and discovery of novel inhibitors against FabD protein of *Mycobacterium tuberculosis* . J. Biomol. Struct. Dyn. 42 (12), 6280–6291. 10.1080/07391102.2023.2233622 37424186

[B136] Sung J. C. Padilla D. J. Garcia-Contreras L. Verberkmoes J. L. Durbin D. Peloquin C. A. (2009). Formulation and pharmacokinetics of self-assembled rifampicin nanoparticle systems for pulmonary delivery. Pharm. Res. 26 (8), 1847–1855. 10.1007/s11095-009-9894-2 19407933 PMC10247220

[B137] Taira J. Umei T. Inoue K. Kitamura M. Berenger F. Sacchettini J. C. (2020). Improvement of the novel inhibitor for mycobacterium enoyl-acyl carrier protein reductase (InhA): a structure-activity relationship study of KES4 assisted by *in silico* structure-based drug screening. J. Antibiot. (Tokyo) 73 (6), 372–381. 10.1038/s41429-020-0293-6 32152525

[B138] Tayal S. Singh V. Bhatnagar S. (2025). 3D-QSAR and ADMET studies of morpholino-pyrimidine inhibitors of DprE1 from *Mycobacterium tuberculosis* . J. Biomol. Struct. Dyn. 43 (6), 2948–2967. 10.1080/07391102.2023.2294496 38112325

[B139] Thompson A. M. Cheung C. Y. McNeil M. B. Campbell A. C. Záhorszká M. Korduláková J. (2026). Advancing the antituberculosis activity of nitropicolinic acids and amides. Eur. J. Med. Chem. 302 (Pt 2), 118324. 10.1016/j.ejmech.2025.118324 41202651

[B140] Tiwari A. P. Sridhar B. Boshoff H. I. Arora K. Gautham Shenoy G. Vandana K. E. (2020). Design, synthesis, *in silico* and *in vitro* evaluation of novel diphenyl ether derivatives as potential antitubercular agents. Mol. Divers. 24 (4), 1265–1279. 10.1007/s11030-019-09990-z 31506871 PMC11177332

[B141] Vieira A. C. Magalhães J. Rocha S. Cardoso M. S. Santos S. G. Borges M. (2017). Targeted macrophages delivery of rifampicin-loaded lipid nanoparticles to improve tuberculosis treatment. Nanomedicine (Lond). 12 (24), 2721–2736. 10.2217/nnm-2017-0248 29119867

[B142] Vieira T. F. Martins F. G. Moreira J. P. Barbosa T. Sousa S. F. (2021). *In silico* identification of possible inhibitors for protein kinase B (PknB) of *Mycobacterium tuberculosis* . Molecules 26 (20), 6162. 10.3390/molecules26206162 34684743 PMC8541300

[B143] Wang H. Li D. Song L. Zhou C. Huang X. Huang W. (2025). Mycobacterial phthiocerol dimycocerosate induces Galectin-3 upregulation to impair proinflammatory responses and favor immune evasion. Int. J. Biol. Macromol. 321 (Pt 1), 146193. 10.1016/j.ijbiomac.2025.146193 40701478 PMC12379081

[B109] World Health Organization (2025). Global Tuberculosis Report 2025. Geneva, Switzerland: World Health Organization.

[B144] Xu J. Zhang M. Xie F. Zhen J. Abuliken Y. Gao C. (2025). Mycobacterium transcriptional factor BlaI regulates cell division and growth and potentiates beta-lactam antibiotic efficacy against mycobacteria. Microorganisms 13 (10), 2245. 10.3390/microorganisms13102245 41156705 PMC12565901

[B145] Yalcin-Ozkat G. Ersan R. H. Ulger M. Ulger S. T. Burmaoglu S. Yildiz I. (2023). Design, synthesis, and computational studies of benzimidazole derivatives as new antitubercular agents. J. Biomol. Struct. Dyn. 41 (7), 2667–2686. 10.1080/07391102.2022.2036241 35132948

[B146] Yan Z. Guo J. Wu J. Zhang H. Ma T. (2025). Generation of novel polyclonal antibodies against *Mycobacterium tuberculosis* lipoarabinomannan, EspB, and Mtb8. Appl. Microbiol. Biotechnol. 109 (1), 200. 10.1007/s00253-025-13588-x 40938391 PMC12431886

[B147] Yang W. Zhao H. Zhao Z. Pei S. Zhu Z. Huang Z. (2025). Insights into anti-tuberculosis drug design on the scaffold of nitroimidazole derivatives using structure-based computer-aided approaches. RSC. Adv. 15 (28), 22745–22763. 10.1039/d5ra01362c 40612639 PMC12224256

[B148] Yao R. Wang B. Fu L. Li L. You K. Li Y. G. (2022). Sudapyridine (WX-081), a novel compound against *Mycobacterium tuberculosis* . Microbiol. Spectr. 10 (1), e0247721. 10.1128/spectrum.02477-21 35170994 PMC8849072

[B149] Zawal A. G. Abdel-Aziz M. M. Elbatreek M. H. El-Shanawani A. A. Abdel-Aziz L. M. Elbaramawi S. S. (2023). Design, synthesis, *in vitro* and *in silico* evaluation of novel substituted 1,2,4-triazole analogues as dual human VEGFR-2 and TB-InhA inhibitors. Bioorg. Chem. 141, 106883. 10.1016/j.bioorg.2023.106883 37774433

[B150] Zhang Y. Wu R. Sun M. Li X. Fang R. Xing J. (2025). Progress of anti-tuberculosis drug targets and novel therapeutic strategies. Front. Microbiol. 16, 1637254. 10.3389/fmicb.2025.1637254 40980328 PMC12446298

[B151] Zhang M. Allen R. Ames L. Engelhart C. A. Quach D. Lv X. (2025). Microbiological evidence for the trisubstituted benzimidazoles targeting MmpL3 in mycobacterium tuberculosis. Antimicrob agents chemother. *Antimicrob. Agents* . Chemother. 69 (10), e0036825. 10.1128/aac.00368-25 PMC12486814 40827962

[B152] Zhou Z. Wattiez R. Constant P. Marrakchi H. Soetaert K. Mathys V. (2023). Telacebec interferes with virulence lipid biosynthesis protein expression and sensitizes to other antibiotics. Microorganisms 11 (10), 2469. 10.3390/microorganisms11102469 37894127 PMC10609169

