# Supplementary material for: Breaking the barrier: from biosynthetic inhibition to multidimensional modulation of the mycobacterial cell wall in tuberculosis therapy
Source: Front Pharmacol. 2026 Jun 15;17:1863735. doi: 10.3389/fphar.2026.1863735 (PMC13311081; doi:10.3389/fphar.2026.1863735)
Supplement: Supplementary file 1 [file Table1.doc]

## Supplementary Table S1. Representative inhibitors of Mtb cell wall biosynthesis with MIC values and Enzyme inhibition

| Compound name | Chemical scaffold / class | Target | MIC (against Mtb) | Enzyme inhibition (IC₅₀ / Ki) | Experimental validation | Reference(s) |
| --- | --- | --- | --- | --- | --- | --- |
| **DprE1 inhibitors** |  |  |  |  |  |  |
| OPC‑167832 | 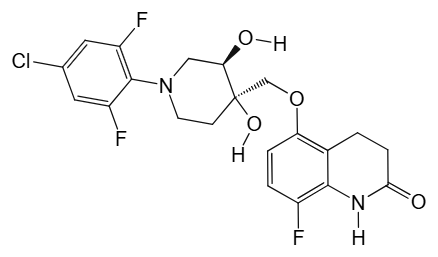 | DprE1 | 0.00024–0.002 μg/mL | IC₅₀ = 0.258 μM | Yes (in vivo) | [1] |
| Compounds 6 (PBTZ169 analogs) | 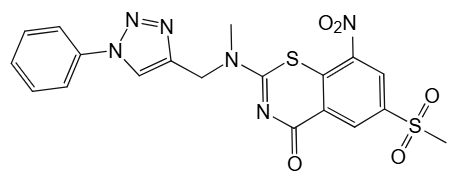 | DprE1 | 47 nM | – | Yes | [2] |
| Compounds 38 (PBTZ169 analogs) | 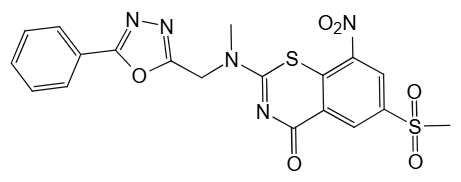 | DprE1 | 30 nM | – | Yes | [2] |
| BTZ‑043 | 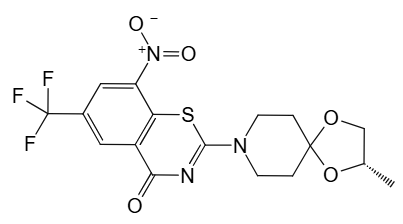 | DprE1 | 0.008 μg/mL | – | Yes (in vivo) | [3,4] |
| MsPBTZ169 | 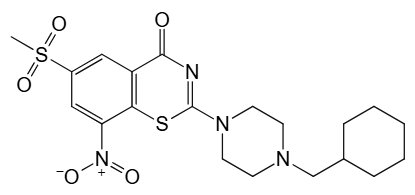 | DprE1 | 0.01 μM | IC₅₀ = 0.05 ± 0.03 μM | Yes | [5] |
| Pyrimidinetrione derivatives (compound 42) | 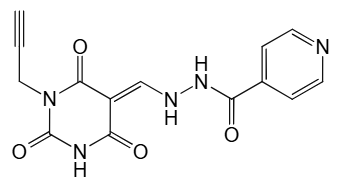 | DprE1 | MIC_50_=1.071±0.041 μM | IC₅₀ = 12.72 μM | Yes | [6] |
| Hydantoin derivatives (compound 30) | 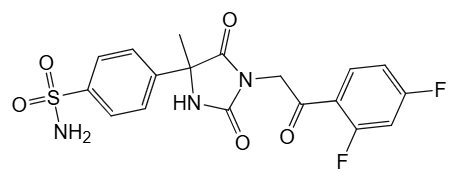 | DprE1 | 0.6μM | IC₅₀ = 0.05 μM | Yes | [7] |
| MP‑38 | 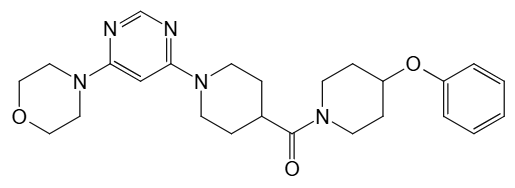 | DprE1 | – | −11.2 kcal/mol | In silico + enzyme assay | [8] |
| ZINC12196803 | 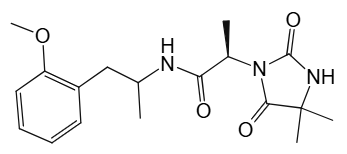 | DprE1 | – | −9.437 kcal/mol | In silico only | [9] |
| Ligand 2 (quinoxaline derivative) | 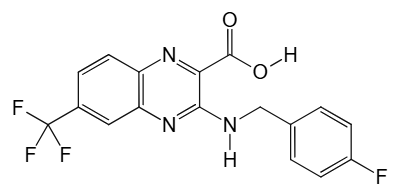 | DprE1 (C387N mutant) | – | −10.6 kcal/mol | In silico only | [10] |
| Benzimidazole derivative 21 | 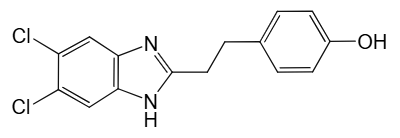 | DprE1 | 7.81μg/mL | –8.5 kcal/mol | In silico only | [11] |
| **DprE2 inhibitors** |  |  |  |  |  |  |
| Pretomanid | 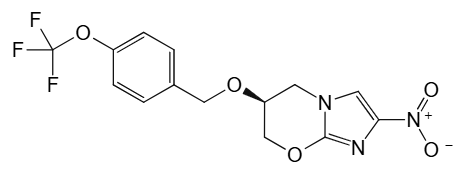 | DprE2 | – | IC₅₀ = 8.3±0.2 μM | Yes | [12] |
| Delamanid | 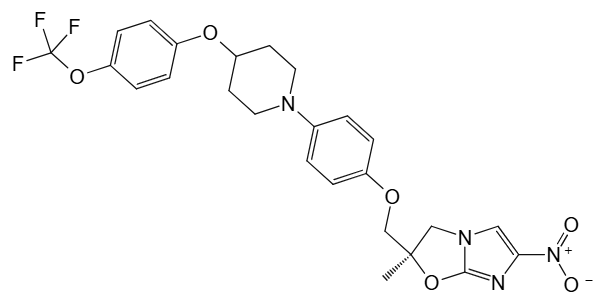 | DprE2 | – | – | Yes | [12] |
| **Arabinosyltransferase & galactan biosynthesis inhibitors** |  |  |  |  |  |  |
| Ethambutol | 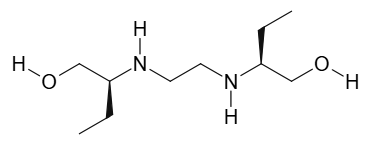 | EmbC | 0.5–2 μg/mL | –4.52 kcal/mol | Yes (approved drug) | [13] |
| Emb1 | 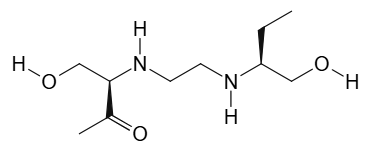 | EmbC | – | −5.77  kcal/mol | In silico only | [13] |
| Emb3 | 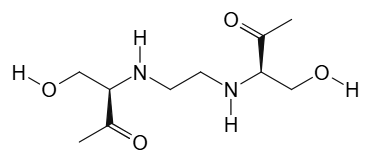 | EmbC | – | −5.13 kcal/mol | In silico only | [13] |
| DA10 (MS208 derivative) | 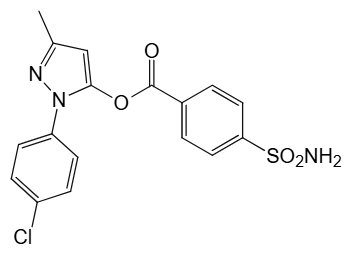 | UGM | ＞100 μg/mL | Ki=51±4 μM | Enzyme assay only | [14] |
| Oxazepino indole(compound 10a) | 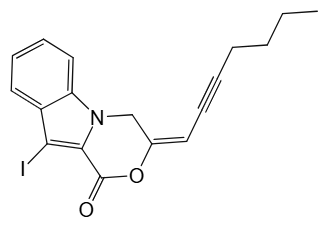 | UGM | 6.2 μg/mL | Kd=66.09 μM | In vitro | [15] |
| **Peptidoglycan synthesis inhibitors** |  |  |  |  |  |  |
| B59 | 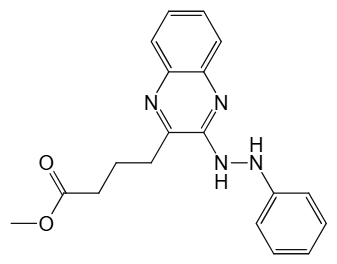 | DapB | MIC₉₉ 20 μg/mL | −8.59  kcal/mol | Yes (enzyme IC₅₀ 11 μg/mL) | [16] |
| Naringenin | **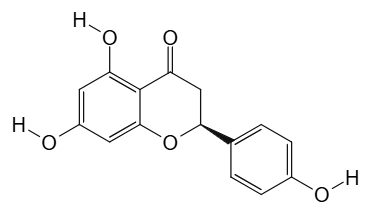** | MurI | – | Ki=23.8 μM | Enzyme assay | [17] |
| Quercetin | 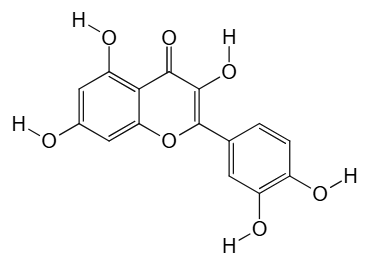 | MurI | – | Ki=20.8 μM | Enzyme assay | [17] |
| Durlobactam | 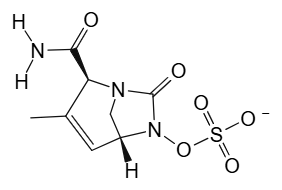 | Ldt + PBP | 0.5-16 μg/mL | Ki=9.2±0.9 μM | Yes | [18] |
| **Mycolic acid biosynthesis inhibitors** |  |  |  |  |  |  |
| Isoniazid | 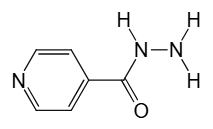 | InhA | 1 μM | – | Yes (approved drug) | [13] |
| Diaryl ether dehydrozingerone (compound 7) | 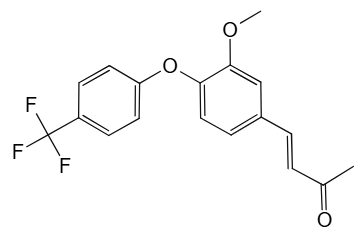 | InhA | MBC=4 μg/mL | −9.6 kcal/mol | Yes (against MDR isolates) | [19] |
| Diaryl ether dehydrozingerone (compound 14) | 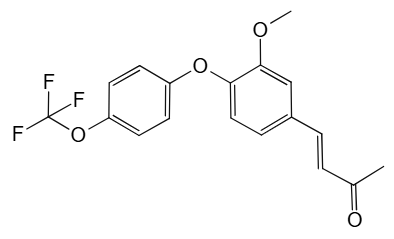 | InhA | MBC=8 μg/mL | −9.3 kcal/mol | Yes (against MDR isolates) | [19] |
| 1,2,4‑Triazole derivatives (6h,6i,6l,11c) | 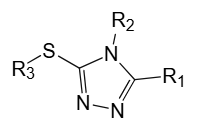 | InhA | – | IC₅₀ =1.3–4.7 μM | Yes | [20] |
| PYN‑8 | 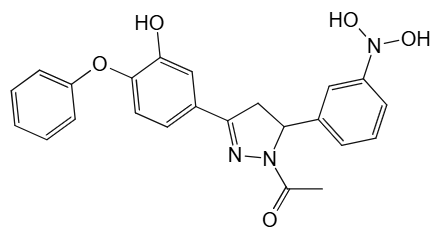 | InhA | 4–7 μM | – | Yes | [21] |
| AN12541 | 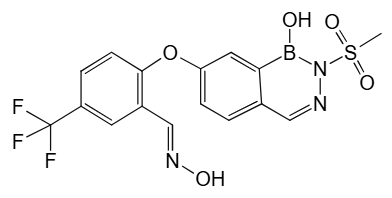 | InhA | – | IC₉₀= 0.11 ± 0.21 μM | Yes | [22] |
| AN12855 | 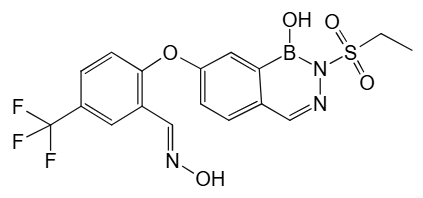 | InhA | – | IC₉₀= 0.090 ± 0.050 μM | Yes | [22] |
| AP‑02 | 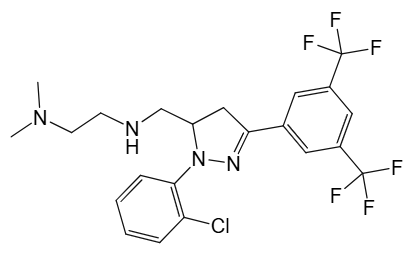 | Ag85C | MIC_99_ = 13μM (against M. smegmatis); MIC_99_ = 25μM (against M. bovis BCG) | – | Yes | [23] |
| AP‑05 | 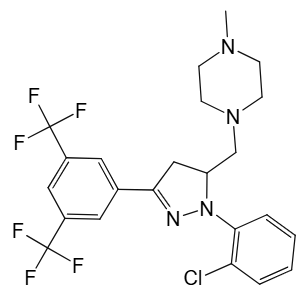 | Ag85C | MIC_99_ = 16μM (against M. smegmatis); MIC_99_ = 20μM (against M. bovis BCG) | – | Yes | [23] |
| iBpPPOX | 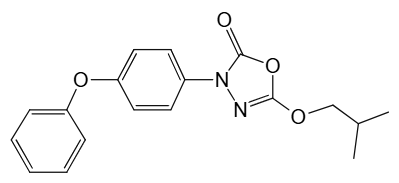 | Ag85C | MIC₅₀ = 33.0 ± 2.0 μM (against M. abscessus) | – | ABPP confirmed | [24] |
| CMX410 | 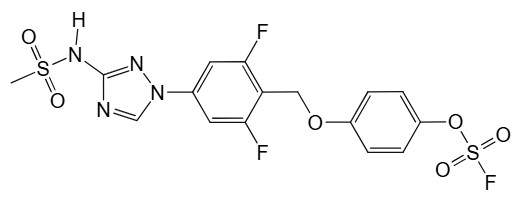 | Pks13 (AT domain) | 0.031μM | – | Yes (oral, in vivo) | [25] |
| BMVC‑8C3O | 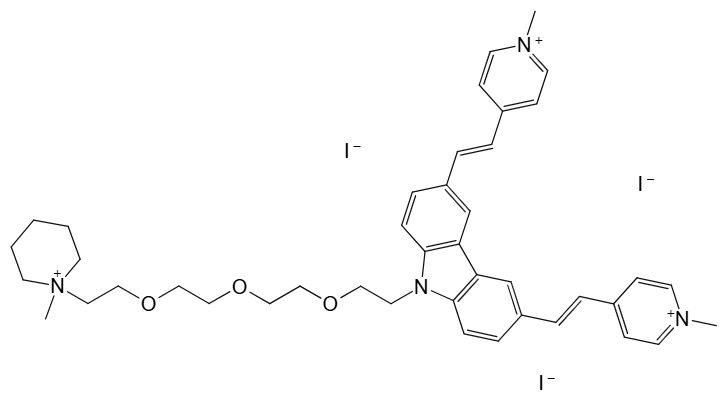 | Pks13 | - | IC₅₀ =6.94μM | SPR, docking | [26] |
| Coumestan derivatives (compound 1) | 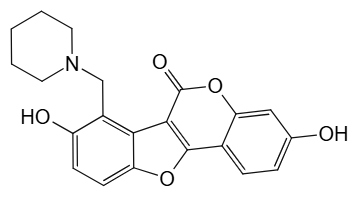 | Pks13 | MBC = 0.0039–0.0078 μg/mL | – | Yes (in vivo) | [27] |
| Closantel | 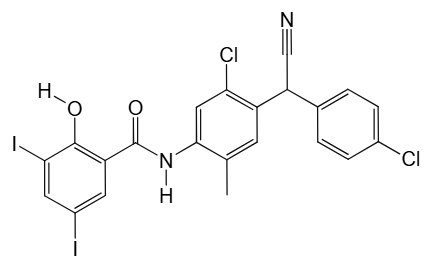 | FadD32 | 0.08 μM | IC₅₀ = 7.7 μM | Thermal shift, docking | [28] |
| M1 | 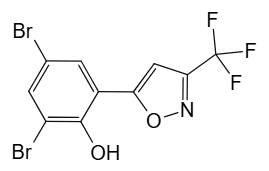 | FadD32, FadD28 | − | IC₅₀ = 4.8 μM | Yes (in vivo) | [29] |
| 2-[(2-amino-6-methylpyrimidin-4-yl)sulfanyl]-N-arylacetamides (Compound 6c) | 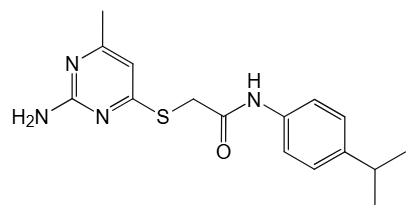 | CmaA1 (predicted) | 1.5μg/mL | −9.8 kcal/mol | Docking only | [30] |
| 2-[(2-amino-6-methylpyrimidin-4-yl)sulfanyl]-N-arylacetamides (Compound 6i) | 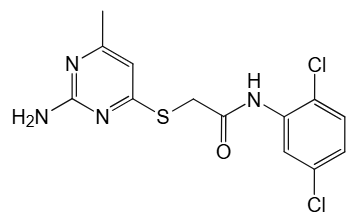 | CmaA1 (predicted) | 1.5μg/mL | −9.1 kcal/mol | Docking only | [30] |
| **MmpL3 inhibitors** |  |  |  |  |  |  |
| SQ109 | 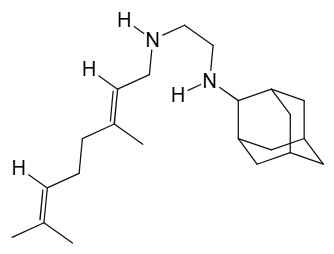 | MmpL3 (also respiration) | 0.25 μg/mL | –13.8 kcal/mol | Yes | [31，32] |
| AU1235 | 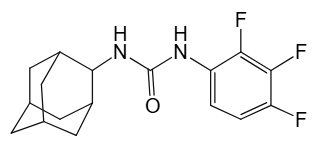 | MmpL3 | 0.3μM | – | Yes | [33] |
| NITD‑349 | 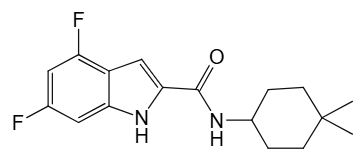 | MmpL3 | MBC=0.125μM | – | Yes | [34] |
| ST004 | 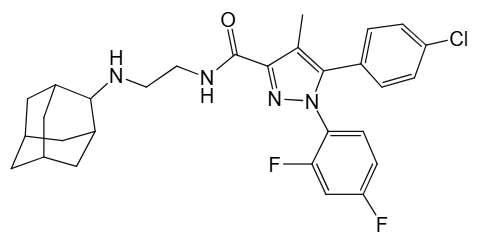 | MmpL3 | 4 μg/mL | – | Cryo‑EM confirmed | [35] |
| TPN‑0157345 | 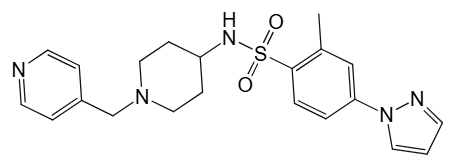 | MmpL3 | 3.3μM | – | BCP, iniB induction | [36] |
| Phenyl urea derivatives (Compound 21) | 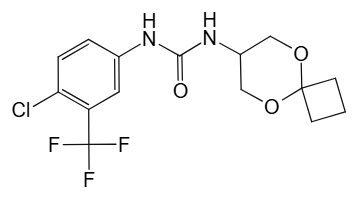 | MmpL3 | 0.78 μM | – | Yes (oral) | [37] |
| Pyrazole amides (Compound 15) | 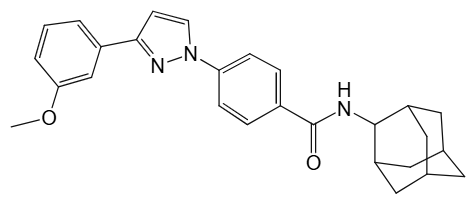 | MmpL3 | 2 μg/mL | – | Docking + genetics | [38] |
| Pyrazole amides (Compound 35) | 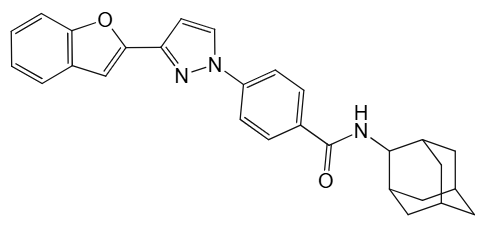 | MmpL3 | 2-4 μg/mL | − | Docking + genetics | [38] |
| Adamantanol derivatives 8j | 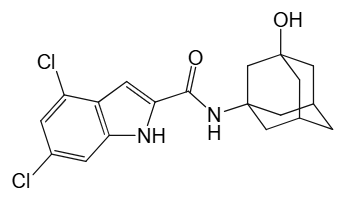 | MmpL3 | 0.66 μM | -12.5- -13.6 kcal/mol | Docking | [39] |
| Adamantanol derivatives 8k | 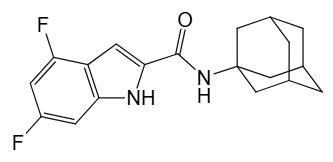 | MmpL3 | 0.012 μM | -12.5- -13.6 kcal/mol | Docking | [39] |
| **Other transport proteins** |  |  |  |  |  |  |
| LB04 | 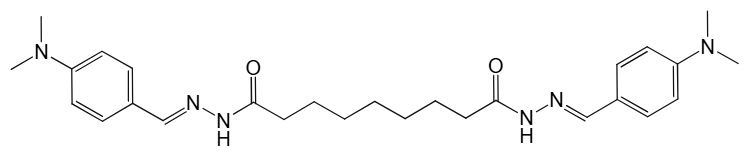 | LprG | − | EC₅₀ ≈ 100 μM (fluorescence displacement assay) | Fluorescence binding | [40] |
| **Cell wall integrity disruptors** |  |  |  |  |  |  |
| RMB041 | 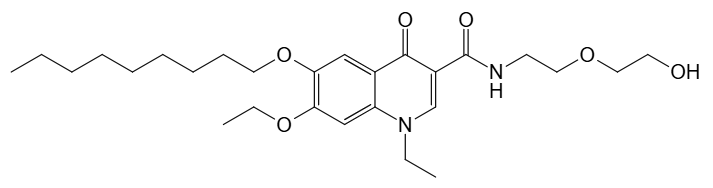 | Cell wall (lipid metabolism) | MIC_90_ = 1.61 µM | – | Metabolomics | [41] |
| Arenicolide Ar‑A | 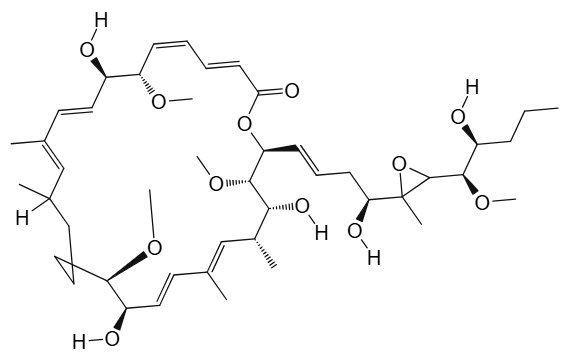 | Cell wall (ATP depletion) | MIC_50_ =0.8 μM | IC₅₀ = 0.9 μM | Yes (zebrafish/mouse) | [42] |
| Tanshinone I | 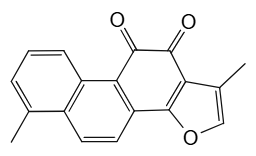 | Cell wall (PE_PGRS) | MIC₉₀=1.03μg/mL | – | SEM, resistance mutants | [43] |
| Tanshinone IIA | 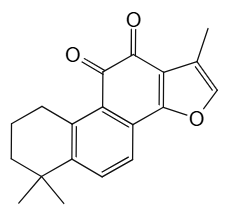 | Cell wall (PE_PGRS) | MIC₉₀=0.38μg/mL | – | SEM, resistance mutants | [43] |
| Cryptotanshinone | 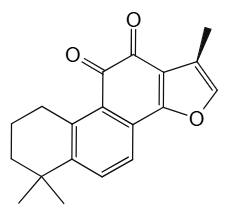 | Cell wall (PE_PGRS) | MIC₉₀=1.21μg/mL | – | SEM, resistance mutants | [43] |
| RapTB | VCVLAHHFGKEFTPPVQAAYQKVVAGVANALAHKYH | Cell wall integrity | 53% inhibition at 50 μM (³H‑uracil assay) | – | Yes | [44] |
| **Energy metabolism inhibitors** |  |  |  |  |  |  |
| Telacebec (Q203) | 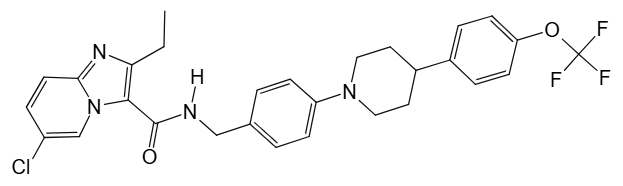 | QcrB (cytochrome bc1) | 5 nM (against M. bovis BCG) | – | Yes | [45,46] |
| WX‑081 (Sudapyridine) | 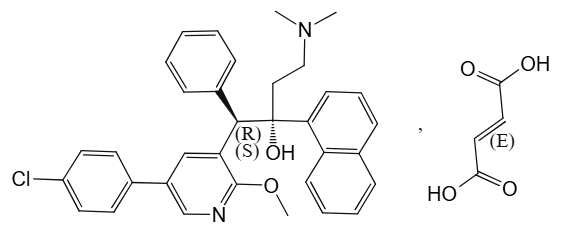 | ATP synthase | 0.07 μg/mL | – | Yes | [47] |

**References**

1. Hariguchi, N., Chen, X., Hayashi, Y., Kawano, Y., Fujiwara, M., Matsuba, M., et al. (2020) OPC-167832, a Novel Carbostyril Derivative with Potent Antituberculosis Activity as a DprE1 Inhibitor. *Antimicrobial. Agents. and. Chemotherapy*. 64(6), e02020-19. doi: 10.1128/AAC.02020-19.
2. Fan, D., Wang, B., Stelitano, G., Savková, K., Shi, R., Huszár, S., et al. (2021). Structural and Activity Relationships of 6-Sulfonyl-8-Nitrobenzothiazinones as Antitubercular Agents. *J. Med. Chem.* 64(19), 14526-14539. doi: 10.1021/acs.jmedchem.1c01049.
3. Ramey, M.E., Kaya, F., Bauman, A.A., Massoudi, L.M., Sarathy, J.P., Zimmerman, M.D., et al. (2023). Drug distribution and efficacy of the DprE1 inhibitor BTZ-043 in the C3HeB/FeJ mouse tuberculosis model. *Antimicrob. Agents. Chemother.* 67(11), e0059723. doi: 10.1128/aac.00597-23.
4. Eckhardt, E., Li, Y., Mamerow, S., Schinköthe, J., Sehl-Ewert, J., Dreisbach, J., et al. (2023). Pharmacokinetics and Efficacy of the Benzothiazinone BTZ-043 against Tuberculous Mycobacteria inside Granulomas in the Guinea Pig Model. *Antimicrob. Agents. Chemother.* 67(4), e0143822. doi: 10.1128/aac.01438-22.
5. Shi, R., Wang, B., Stelitano, G., Wu, X., Shan, Y., Wu, Y., et al., (2022). Development of 6-Methanesulfonyl-8-nitrobenzothiazinone Based Antitubercular Agents. *ACS. Med. Chem. Lett.* 13(4), 593-598. doi: 10.1021/acsmedchemlett.1c00652.
6. Liang, J., Liu, Y., Guan, Q., Li, Y., Zheng, M.Z., Zhang, X.L., et al. (2025). Discovery of novel pyrimidinetrione derivatives as DprE1 inhibitors with potent antimycobacterial activities. *Eur. J. Med. Chem.* 289, 117416. doi: 10.1016/j.ejmech.2025.117416.
7. Balabon, O., Pitta, E., Rogacki, M.K., Meiler, E., Casanueva, R., Guijarro, L., et al. (2020). Optimization of Hydantoins as Potent Antimycobacterial Decaprenylphosphoryl-β-d-Ribose Oxidase (DprE1) Inhibitors. *J. Med. Chem.* 63(10), 5367-5386. doi: 10.1021/acs.jmedchem.0c00107.
8. Tayal, S., Singh, V., and Bhatnagar, S. (2025). 3D-QSAR and ADMET studies of morpholino-pyrimidine inhibitors of DprE1 from Mycobacterium tuberculosis. *J. Biomol. Struct. Dyn*. 43(6), 2948-2967. doi: 10.1080/07391102.2023.2294496.
9. Mali, S.N., Pandey, A., Bhandare, R.R., and Shaik, A.B. (2022). Identification of hydantoin based Decaprenylphosphoryl-β-d-Ribose Oxidase (DprE1) inhibitors as antimycobacterial agents using computational tools. *Sci. Rep.* 12(1), 16368. doi: 10.1038/s41598-022-20325-1.
10. El Haddoumi, G., Mansouri, M., Kourou, J., Belyamani, L., Ibrahimi, A., and Kandoussi, I. (2024). Targeting decaprenylphosphoryl-β-D-ribose 2′-epimerase for Innovative Drug Development Against Mycobacterium Tuberculosis Drug-Resistant Strains. *Bioinform. Biol. Insights*. 18, 11779322241257039. doi: 10.1177/11779322241257039.
11. Yalcin-Ozkat, G., Ersan, R.H., Ulger, M., Ulger, S.T., Burmaoglu, S., Yildiz, I., et al. (2023). Design, synthesis, and computational studies of benzimidazole derivatives as new antitubercular agents. *J. Biomol. Struct. Dyn*. 41(7), 2667-2686. doi: 10.1080/07391102.2022.2036241.
12. Abrahams, K.A., Batt, S.M., Gurcha, S.S., Veerapen, N., Bashiri, G., and Besra, G.S. (2023). DprE2 is a molecular target of the anti-tubercular nitroimidazole compounds pretomanid and delamanid. *Nat. Commun.* 14(1), 3828. doi: 10.1038/s41467-023-39300-z.
13. Das, N., Jena, P.K., and Pradhan, S.K. (2020). Arabinosyltransferase C enzyme of Mycobacterium tuberculosis, a potential drug target: An insight from molecular docking study. *Heliyon.* 6(2), e02693. doi: 10.1016/j.heliyon.2019.e02693.
14. Ahmed, D.M., Chen, J.M., and Sanders, D.A.R. (2022). Sanders, Pyrazole and Triazole Derivatives as Mycobacterium tuberculosis UDP-Galactopyranose Inhibitors. *Pharmaceuticals (Basel)*. 15(2), 197. doi: 10.3390/ph15020197.
15. Maaliki, C., Fu, J., Villaume, S., Viljoen, A., Raynaud, C., Hammoud, S., et al. (2020). Synthesis and evaluation of heterocycle structures as potential inhibitors of Mycobacterium tuberculosis UGM. *Bioorg. Med. Chem.* 28(13), 115579. doi: 10.1016/j.bmc.2020.115579.
16. Angrish, N., Lalwani, N., and Khare, G. (2023). In silico virtual screening for the identification of novel inhibitors against dihydrodipicolinate reductase (DapB) of Mycobacterium tuberculosis, a key enzyme of diaminopimelate pathway. *Microbiol. Spectr*. 11(6), e0135923. doi: 10.1128/spectrum.01359-23.
17. Pawar, A., Jha, P., Chopra, M., Chaudhry, U., and Saluja, D. (2020). Screening of natural compounds that targets glutamate racemase of Mycobacterium tuberculosis reveals the anti-tubercular potential of flavonoids. *Sci. Rep*. 10(1), 949. doi: 10.1038/s41598-020-57658-8.
18. Nantongo, M., Nguyen, D.C., Bethel, C.R., Taracila, M.A., Li, Q., Dousa, K.M., et al. (2024). Durlobactam, a Diazabicyclooctane β-Lactamase Inhibitor, Inhibits BlaC and Peptidoglycan Transpeptidases of Mycobacterium tuberculosis. *ACS. Infect. Dis*. 10(5), 1767-1779. doi: 10.1021/acsinfecdis.4c00119.
19. Mubarak, M.M., Kantroo, H.A., Mir, F.A., Kumar, C., and Ahmad, Z. (2025). Targeting InhA in drug-resistant Mycobacterium tuberculosis: potent antimycobacterial activity of diaryl ether dehydrozingerone derivatives. *Arch. Microbiol.* 207(2), 34. doi: 10.1007/s00203-025-04238-x.
20. Zawal, A.G., Abdel-Aziz, M.M., Elbatreek, M.H., El-Shanawani, A.A., Abdel-Aziz, L.M., and Elbaramawi, S.S. (2023). Design, synthesis, in vitro and in silico evaluation of novel substituted 1,2,4-triazole analogues as dual human VEGFR-2 and TB-InhA inhibitors. *Bioorg. Chem*. 141, 106883. doi: 10.1016/j.bioorg.2023.106883.
21. Tiwari, A.P., Sridhar, B., Boshoff, H.I., Arora, K., Gautham Shenoy, G., Vandana, K.E., et al. (2020). Design, synthesis, in silico and in vitro evaluation of novel diphenyl ether derivatives as potential antitubercular agents. *Mol. Divers.* 24(4), 1265-1279. doi: 10.1007/s11030-019-09990-z.
22. Flint, L., Korkegian, A., and Parish, T. (2020). InhA inhibitors have activity against non-replicating Mycobacterium tuberculosis. *PLoS. One.* 15(11), e0239354. doi: 10.1371/journal.pone.0239354.
23. Cui, Y., Lanne, A., Avula, S., Hama Salih, M.A., Peng, X., Milne, G., et al. (2025). Discovery of novel fluorescent amino-pyrazolines that detect and kill Mycobacterium tuberculosis. *Eur. J. Med. Chem.* 297, 117889. doi: 10.1016/j.ejmech.2025.117889.
24. Madani, A., Mallick, I., Guy, A., Crauste, C., Durand, T., Fourquet, P., et al. (2020). Dissecting the antibacterial activity of oxadiazolone-core derivatives against Mycobacterium abscessus. *PLoS. One*. 15(9), e0238178. doi: 10.1371/journal.pone.0238178.
25. Krieger, I.V., Sukheja, P., Yang, B., Tang, S., Selle, D., Woods, A., et al. (2025). SuFEx-based antitubercular compound irreversibly inhibits Pks13. *Nature*. 645(8081), 755-763. doi: 10.1038/s41586-025-09286-3.
26. Liu, T., Meng, J., Wang, B., Li, X., Wang, Q., Liu, S., et al. (2025). Identification of BMVC-8C3O as a novel Pks13 inhibitor with anti-tuberculosis activity. *Tuberculosis (Edinb)*. 150, 102579. doi: 10.1016/j.tube.2024.102579.
27. Lun, S., Xiao, S., Zhang, W., Wang, S., Gunosewoyo, H., Yu, L.F., et al. (2023). Therapeutic potential of coumestan Pks13 inhibitors for tuberculosis. *Antimicrob. Agents. Chemother*. 95(5), e02190-20. doi: 10.1128/AAC.02190-20.
28. Le, N.H., Constant, P., Tranier, S., Nahoum, V., Guillet, V., Maveyraud, L., et al. (2022). Drug screening approach against mycobacterial fatty acyl-AMP ligase FAAL32 renews the interest of the salicylanilide pharmacophore in the fight against tuberculosis. *Bioorg. Med. Chem*. 71, 116938. doi: 10.1016/j.bmc.2022.116938.
29. Rani, N., Rajmani, R.S., and Surolia, A. (2025). Identification of an Isoxazole Derivative as an Antitubercular Compound for Targeting the FadD Enzymes of Mycobacterium tuberculosis. *J. Med. Chem*. 68(1), 270-286. doi: 10.1021/acs.jmedchem.4c01844.
30. Erkin, A.V., Serebryakov, E.B., and Krutikov, V.I. 2-[(2-Amino-6-methylpyrimidin-4-yl)sulfanyl]-N-arylacetamides: Discovery of a new class of anti-tubercular agents and prospects for their further structural modification. B*ioorg. Med. Chem. Lett*. 83, 129189. doi: 10.1016/j.bmcl.2023.129189.
31. Johnson, W.C., Alivisatos, A., Smith 2^nd^, T.C., Van, N., Soni, V., Wallach, J.B., et al. (2025). Integration of multi-modal measurements identifies critical mechanisms of tuberculosis drug action. *Cell, Syst*. 16(8), 101348. doi: 10.1016/j.cels.2025.101348.
32. Han, X., Chen, C., Wang, H., Kang, J., Yan, Q., Ma, Y., et al. (2022). GlmU inhibitor from the roots of Euphorbia ebracteolata as an anti-tuberculosis agent. *RSC. Adv*. 12(28), 18266-18273. doi: 10.1039/d2ra02044k.
33. Madacki, J., Kopál, M., Jackson, M., and Korduláková, J. (2021). Mycobacterial Epoxide Hydrolase EphD Is Inhibited by Urea and Thiourea Derivatives. *Int. J. Mol. Sci.* 22(6), 2884. doi: 10.3390/ijms22062884.
34. Berube, B., Deshpande, A., Bhagwat, A., and Parish, T. (2023). Inoculum-dependent bactericidal activity of a Mycobacterium tuberculosis MmpL3 inhibitor. *Microbiology (Reading)*, 169(6), 001345. doi: 10.1099/mic.0.001345.
35. Xu, J., Zhang, M., Xie, F., Zhen, J., Abuliken, Y., Gao, C., et al. (2025). Mycobacterium Transcriptional Factor BlaI Regulates Cell Division and Growth and Potentiates beta-Lactam Antibiotic Efficacy Against Mycobacteria. *Microorganisms*. 13(10), 2245. doi: 10.3390/microorganisms13102245.
36. Allen, R., Ames, L., Baldin, V.P., Butts, A., Henry, K.J., Durst, G., et al. (2024). An arylsulfonamide that targets cell wall biosynthesis in Mycobacterium tuberculosis. *Antimicrob. Agents. Chemother*. 68(11), e0103724. doi: 10.1128/aac.01037-24.
37. Mostert, D., Braun, J., Zimmerman, M.D., Engelhart, C.A., Berndl, S., Quoika, P.K., et al. (2025). Tailored phenyl ureas eradicate drug-resistant Mycobacterium tuberculosis by targeting mycolic acid cell wall assembly. *Chem. Sci*. 16(21),9472-9483. doi: 10.1039/d5sc02565f.
38. Maddipatla, S., Agnivesh, P.K., Bakchi, B., Nanduri, S., Kalia, N.P., and Yaddanapudi, V.M. (2025). New pyrazole-based derivatives targeting MmpL3 transporter in Mycobacterium tuberculosis: design, synthesis, biological evaluation and molecular docking studies. *Mol. Divers*. 29(6), 6437-6458. doi: 10.1007/s11030-025-11152-3.
39. Alsayed, S.S.R., Lun, S., Payne, A., Bishai, W.R., and Gunosewoyo, H. (2021). Design, synthesis and antimycobacterial evaluation of novel adamantane and adamantanol analogues effective against drug-resistant tuberculosis. *Bioorg. Chem*. 106, 104486. doi: 10.1016/j.bioorg.2020.104486.
40. Bai, L., Parkin, L.A., Zhang, H., Shum, R., Previti, M.L., and Seeliger, J.C. (2020). Dimethylaminophenyl Hydrazides as Inhibitors of the Lipid Transport Protein LprG in Mycobacteria. *ACS. Infect. Dis*. 6(4), 637-648. doi: 10.1021/acsinfecdis.9b00497.
41. Knoll, K.E., Lindeque, Z., Adeniji, A.A., Oosthuizen, C.B., Lall, N., and Loots, D.T. (2021). Elucidating the Antimycobacterial Mechanism of Action of Decoquinate Derivative RMB041 Using Metabolomics. *Antibiotics (Basel)*. 10(6), 693. doi: 10.3390/antibiotics10060693.
42. Hwang, S., Heo, B.E., Nguyen, T.Q., Kim, Y.J., Lee, S.G., Huynh, T.H., et al. (2025). Arenicolide Family Macrolides Provide a New Therapeutic Lead Combating Multidrug-Resistant Tuberculosis. Angew. *Chem. Int. Ed. Engl*. 64(1), e202412994. doi: 10.1002/anie.202412994.
43. Polinário, G., Rosa, M.A.B.C., Campos, D.L., Moraes, L.L.S., de Campos, M.M.A., Silva, I.G.M., et al. (2025). Tanshinones target drug-resistant tuberculosis: efficacy, selectivity, and potential mechanism of action. *RSC. Med. Chem*. 16(12), 6020–30. doi: 10.1039/d5md00637f.
44. Klevesath, L., Noschka, R., Vomhof, T., Mohnani, J., Grieshober, M., Michaelis, J., et al. (2025). RapTB: a lung-derived hemoglobin fragment with activity against Mycobacterium tuberculosis. *Front. Microbiol*. 16, 1669022. doi: 10.3389/fmicb.2025.1669022.
45. Malík, I., Čižmárik, J., Kováč, G., Pecháčová, M., and Hudecova, L. (2021). Telacebec (Q203): Is there a novel effective and safe anti-tuberculosis drug on the horizon? *Ceska. Slov. Farm*. 70(5), 164–171. doi: 10.5817/CSF2021-5-164.
46. Zhou, Z., Wattiez, R., Constant, P., Marrakchi, H., Soetaert, K., Mathys, V., et al. (2023). Telacebec Interferes with Virulence Lipid Biosynthesis Protein Expression and Sensitizes to Other Antibiotics. *Microorganisms*. 11(10), 2469. doi: 10.3390/microorganisms11102469.
47. Yao, R., Wang, B., Fu, L., Li, L., You, K., Li, Y.G., and Lu, Y. (2022). Sudapyridine (WX-081), a Novel Compound against Mycobacterium tuberculosis. *Microbiol. Spectr*. 10(1), e0247721. doi: 10.1128/spectrum.02477-21.
